# Revealing the widespread potential of forests to increase low level cloud cover

**DOI:** 10.1038/s41467-021-24551-5

**Published:** 2021-07-15

**Authors:** Gregory Duveiller, Federico Filipponi, Andrej Ceglar, Jędrzej Bojanowski, Ramdane Alkama, Alessandro Cescatti

**Affiliations:** 1 European Commission Joint Research Centre, Ispra (VA), Italy; 2 Remote Sensing Centre, Institute of Geodesy and Cartography, Warsaw, Poland; 3 Present Address: Max Planck Institute for Biogeochemistry, Jena, Germany; 4 Present Address: Institute for Environmental Protection and Research (ISPRA), Roma, Italy

**Keywords:** Ecosystem services, Climate-change mitigation, Forestry

## Abstract

Forests play a key role in humanity’s current challenge to mitigate climate change thanks to their capacity to sequester carbon. Preserving and expanding forest cover is considered essential to enhance this carbon sink. However, changing the forest cover can further affect the climate system through biophysical effects. One such effect that is seldom studied is how afforestation can alter the cloud regime, which can potentially have repercussions on the hydrological cycle, the surface radiation budget and on planetary albedo itself. Here we provide a global scale assessment of this effect derived from satellite remote sensing observations. We show that for 67% of sampled areas across the world, afforestation would increase low level cloud cover, which should have a cooling effect on the planet. We further reveal a dependency of this effect on forest type, notably in Europe where needleleaf forests generate more clouds than broadleaf forests.

## Abstract (web summary)

Forests can influence climate by affecting low cloud formation, but where and when this occurs is not well known. Here, the authors provide a global-scale assessment, based on satellite remote sensing observations, suggesting afforestation mostly increases low cloud cover which could potentially cool surface temperatures.

## Introduction

Forests provide a multitude of essential services to human society. These include the capture, storage and regulation of water flows, the improvement of air and water quality, the supply of timber, energy and erosion control, the provision of habitat for biodiversity and livelihoods for humans. There is a particular service that forests have that has come to prominent attention in recent years, and it relates to their interactions with the climate system and their climate change mitigation potential^1^.

To avoid the more dire consequences of climate change, humankind not only needs to stop emitting greenhouse gases into the atmosphere, but the excess carbon that has already been emitted must also be removed^2^. Currently, the most realistic technique to effectively capture carbon (among what are known as negative emission technologies or NETs) is to have it sequestered by trees^3,4^. However, to reach the necessary mitigation targets this effectively requires expanding the land covered by forests^3,4^ by means of ambitious forest restoration or afforestation programmes^5,6^. Along this line, various large-scale efforts in planting trees have recently emerged^7^, including the Sub-Saharan Great Green Wall^8^, the Ant Forest in China^9^, and the extensive afforestation programme foreseen under the European Green Deal^10^. Yet planting trees is not a simple solution and can have various climate impacts and mitigation efficacy depending on how and where they are planted^7,11^. Simplistic approaches to tree restoration that do not properly account for the complexities of the plant–atmosphere interactions may underestimate the amount of land needed and overall provide a dangerous and misleading message^12–15^. Ultimately, it is becoming increasingly evident that land-based climate mitigation through afforestation, forest restoration or avoided deforestation should be based on the comprehensive assessment of both the biogeochemical and the biophysical processes triggered by forest cover change ^16^.

Planting trees also affects climate by other means than carbon sequestration. Complex and non-linear forest–atmosphere interactions can dampen or amplify anthropogenic climate change^1,17^. For instance, by having access to more ground water with their deeper rooting system, trees can sustain higher transpiration rates during droughts compared to nearby grasslands or croplands, contributing to the mitigation of heatwaves^18^. On the other hand, their comparatively lower albedo can generate warming to an extent that may even offset their carbon sequestration mitigation potential at higher latitudes, where the difference between dark conifer trees and neighbouring snow-covered open land is large^19^. Depending on which of these different forces prevails, the local net effect of vegetation cover change on land surface temperature can change geographically and seasonally^1^, as demonstrated by recent global studies based on satellite remote sensing observations^20–22^. Such observation-driven analyses are currently helping to evaluate^23,24^ and improve^25^ land surface models, and they are further inspiring the design of tools for assessing land-based climate policies^26^.

Forest cover change can further have indirect biophysical effects on the climate. These are indirect because they do not stem from changes in the properties of the surface directly, but rather from the effect the surface has on the atmospheric boundary layer (ABL) above it. The best example is the increased formation of low-level convective clouds above forests^27–31^. Increased cloud cover can further affect the water cycle by triggering an increase of precipitation^32,33^. The fact that Earth’s vegetation cover can affect rainfall has been known for a long time^27^. The Amazon forest generates about half of its own rainfall by recycling moisture lost through evaporation^34^. This effect can also go beyond trees: high-yielding corn planted in the US Corn Belt was shown to alter the regional climate patterns by inducing cloud formation and thus generating more rain in summer^35^. By modifying the light environment and tempering the build up of heat, the formation of boundary layer cumulus clouds above forests further appears to provide favourable conditions for carbon uptake^29^. But the radiative consequences may be more profound at planetary level. Theoretical work has shown that increases in evaporation (as would occur after considerable afforestation of large parts of the world) can increase low-elevation cloudiness, which increases the planetary albedo to the point of cooling the whole Earth system^36^.

Clouds remain a very sensitive and uncertain component of the climate system^37–39^. They indeed play an important role in regulating Earth’s radiative budget by modulating the amount of energy reflected, emitted and absorbed both at the surface and in the atmosphere^37^. Different types of clouds have different effects on both surface and planetary energy balance: low clouds typically have a cooling effect by reflecting radiation back into space, while high clouds tend to have a warming effect as they trap radiation and emit it back to the surface^40^. On average, clouds exert a net cooling effect on climate, with a large contribution of the low-level cloud^41^. The radiative effects of low-level clouds could further affect deep-convection, influencing the tropics-wide circulation and precipitation^42^. The generation of low clouds follows the day–night cycle of solar flux. During daytime the destabilization of the boundary layer by solar heating of the surface drives convection and forms cumuliform clouds. At night the boundary layer cools, eventually causing condensation to form stratiform clouds, which cool down by emitting longwave radiation back into space^43^. Over land, the clouds with more pronounced diurnal cycles are cumuliform clouds, they seem to be the dominant cloud type over most areas during spring and summer, and they peak invariably around 14:00 in the afternoon^43^.

What are the underlying mechanisms explaining the interplay between clouds and tree canopies? Forests have larger net radiation and surface roughness than open lands, favouring a better exchange of heat, moisture and momentum with the atmosphere, which in turn can generate a stronger convection and a deeper ABL^44,45^. The co-occurrence of an effective lifting mechanism (provided by the sensible heat flux) and sufficient water vapour (provided by the latent heat flux) support the generation of convective clouds in the lower troposphere^31^. Furthermore, research suggests that, thanks to a feedback between cloud generation and its subsequent effect on the incoming radiation, forests tend to foster the establishment of an equilibrium between the temperature and moisture tendencies ensuring that these cloud-forming conditions endure^46^. Cloud formation is further affected by several other vegetation properties such as the access to deep soil water^47^, landscape fragmentation^48,49^ and even by the emissions of biogenic volatile organic compounds (BVOCs)^50^. BVOCs are complex molecules that plants (and trees in particular) produce to regulate various processes such as plant growth, reproduction and defence, but they also rapidly oxidize in the atmosphere into secondary organic aerosols that can grow into cloud condensation nuclei^51^. The complexity of these multiple processes affecting the interplay between forests and cloud formation partly explains why observational evidence on the impact of land use can disagree in magnitude and sign depending on the season and the region of the world under investigation^31,49,52^.

Given this substantial uncertainty in how and where forest can influence cloud formation, there is a compelling case to address a comprehensive global assessment that can guide land-based mitigation strategies of afforestation or forest restoration. This is especially critical as the indirect effect of cloud formation can potentially offset the direct biophysical effects of land cover change^53^. Process-oriented Earth system models are theoretically the ideal tools to reach this goal, as they attempt to represent all land–atmosphere interactions in a self-consistent way. However, models still struggle to represent adequately some processes linked to cloud formation, and they are also limited by the computational difficulty to finely represent these complex biophysical effects at kilometric scale, which would be the reasonable spatial resolution to address realistic land-based mitigation strategies. Satellite remote sensing technology offers an alternative path, as it can effectively provide synchronous estimations of both cloud and land cover in a spatially and temporally consistent way. Unlike with models, there is a limit to how much process-understanding can be made based solely on remote sensing measurements, since not all land–atmospheric processes can be fully observed. However, this technique offers a unique opportunity to study the effects of land cover change on cloud cover in a consistent manner across seasonal and latitudinal gradients.

In this study we make a global-scale observational assessment of where afforestation could lead to an increased cloud formation by exploiting satellite data products. Our study shows that afforestation generally leads to an increase in low cloud cover over most of the world, and predominantly in the warmer months of the years. These results are comforted by a series of ancillary analyses using either an alternative methodology, a finer scale dataset or even ground-based observations. We further found that different types of forests can have stronger effects on low-level cloud formation. We expect this work to serve as a valuable observational benchmark for climate models, which should improve our general understanding of the Earth System, and also to guide the design of ambitious nature-based mitigation policies such as the European Green Deal.

## Results and discussion

### Global patterns of cloud formation

To characterize the potential effect that afforestation could have on low convective cloud formation, the main approach we use relies on a substitution of space-for-time over a local moving window. As explained in detail in the ‘Methods’ section, we are able to isolate this local effect of low-level cloud formation by systematically comparing neighbouring observations of cloud occurrence over locally contrasting landscapes, whilst ensuring observations are made at the time of peak convective cloud formation to limit effects of lateral advection, and carefully avoiding topographical effects. The approach relies on combining climate data records of land cover^54^ and cloud fractional cover (CFrC) derived from a climatology of satellite observations covering the period 2004–2014^55^. The work is done at monthly temporal scale and with a moving window approach consisting of 7 × 7 cells, each with a spatial resolution of 0.05° (see ‘Methods’ section for details).

**Fig. 1:**
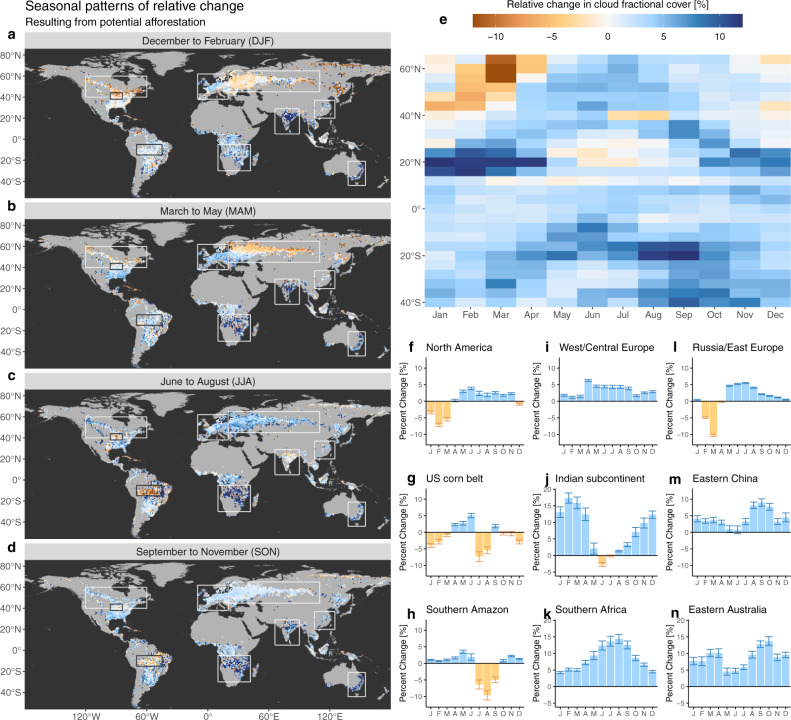
Changes in cloud fractional cover (CFrC) following potential afforestation as derived from satellite data, expressed in relative terms with respect to the average cloudiness over every grid cell. **a**–**d** Maps of seasonal patterns (aggregated to a reduced spatial resolution of 1° for visualization purposes). **e** Latitudinal averages. **f**–**n** Seasonal behaviour of the mean change in CFrC over selected regions, each bar representing a month from January until December. The error bars represent the standard error around the mean. The grey areas in the maps represent places where no estimations are possible due to insufficient coverage of either forests or low vegetation. The same figure is available for the absolute changes in CFrC in the Supplementary material.

An overview of the first comprehensive and observation-based global assessment of the effect of forests on low cloud cover is presented in Fig. 1. While some areas can locally present a certain degree of spatial variability, as could be expected given the necessary assumptions needed to isolate a process such as cloud formation (see ‘Methods’ section for details), there are remarkable macro-patterns of spatial consistency across large geographic areas. It is important to note that, by design, the methodology to extract these local changes in CFrC is essentially sensitive to low-level clouds, and more precisely to boundary layer cumulus clouds (see ‘Methods’ section for details). Therefore, all changes shown in CFrC should be considered as changes in low-level convective clouds only. The spatial coverage in Fig. 1 is restricted to areas where there is sufficient local co-occurrence of both forests and low vegetation cover (e.g. crops and grasslands) for the method to be applicable. Results are shown for transitions from low vegetation towards forests, in order to represent the effect of potential afforestation and thus stay in the context of land-based mitigation. The change is expressed in relative terms with respect to the average cloud cover over each grid cell, as provided by the input cloud product. The absolute change in CFrC is provided in a similar figure in the Supplementary material (Supplementary Fig. 1).

**Fig. 2:**
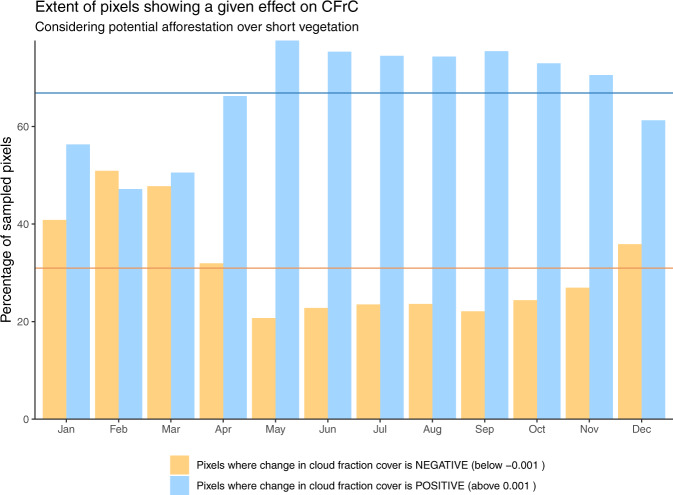
Seasonal patterns of the percentage of sampled pixels where forest has a positive (blue) or negative (yellow) influence on the cloud fractional cover (CFrC). For the Southern Hemisphere, the months have been cycled by 6 months. The horizontal lines represent the annual average.

Overall, we see that for 67% of the area we sampled across the world, extending forest cover is expected to result in an increase in cloud cover. Figure 2 shows how this number rises above 74% during the period from May to September (after cycling the months in the Southern Hemisphere by 6 months to align the seasons according to incoming sunlight). Most parts of the world display clear seasonal patterns of cloud formation following potential afforestation (Fig. 1a–e). The boreal summer is characterized by a relatively strong and consistent increase of local CFrC over forests of the order of about 5% of the normal cloud fraction encountered at grid level, which in relative terms consists of an increase of 0.03 of CFrC. However, during boreal winter and spring across large parts of North America, (Fig. 1f), Russia and Eastern Europe (Fig. 1l), corresponding to areas with prolonged snow cover, the signal is inverted, meaning that there are less clouds over forest than over open land. Temperate regions, which do not have such long snow-covered periods, generally show positive increases in CFrC throughout the year with milder changes in winter (e.g. West/Central Europe in Fig. 1i). We do see various regions that display a characteristic inversion in the sign of CFrC change during periods and places where there is no snow (see Supplementary Fig. 3 for details), including the US Corn Belt in summer (Fig. 1g), the Indian subcontinent during the early monsoon season (Fig. 1j) and the Southern part of the Amazon basin during the drier season (Fig. 1h). Finally, there are also other places with constantly positive differences in CFrC all along the year with a marked seasonal cycle. For some regions, namely India and Southern Africa, the increase in relative terms can be strongly seasonal, with values reaching as high as 15% (Fig. 1j, k). Eastern China (Fig. 1m) shows a strong contrast between a late spring with low-peak and a late summer with high-peak. Drier regions characterized with a combination of high air temperature and high radiation load, such as Southern Africa (Fig. 1k) and Eastern Australia (Fig. 1g), seem to constantly have more clouds above forests.

**Fig. 3:**
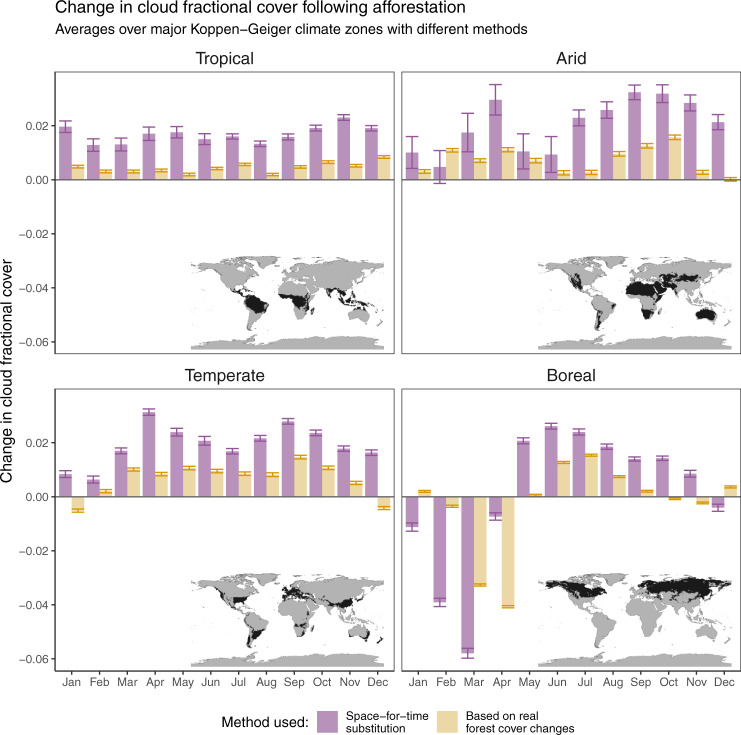
Comparison between different methods to extract the change in cloud fractional cover (CFrC) following afforestation from satellite records. The seasonality of the Southern Hemisphere was shifted by 6 months to align it with that on the northern one. The error bars represent the standard error around the mean.

**Fig. 4:**
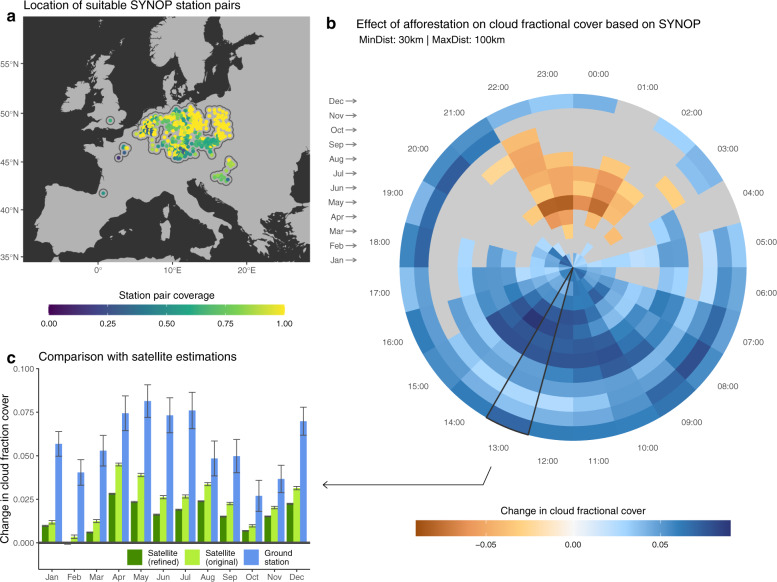
Changes in cloud fractional cover (CFrC) following afforestation as estimated from paired ground stations. **a** Location of the usable pairs of SYNOP ground stations after selecting those where the distance among the pairs is between 30 and 100 km, and which report a sufficiently dense observation coverage during the period 2004–2014. The coverage reported in the colour bar represents the fraction of the bins in (**b**) to which the station contributes. **b** Average change in CFrC after potential afforestation for the region declined by month and hour. Sectors of the disk in grey represent those where the difference in not greater than two times the standard error (note that the colour bar here extends to higher values that similar bars in the rest of the figures). **c** Direct comparison with satellite observations for the time slice nearest to the satellite overpass time (circa 13:30 at the equator). The value of the satellite estimates, for both the refined (0.02°) and the original (0.05°) products, is the mean of all records collected based on a buffer of 50 km around the ground stations, and which is portrayed in (**a**). The error bars represent one standard error around the mean.

Several lines of experimental evidence confirm that the cloud signal we extract is indeed related to potential forest cover change, and that it does not originate from possible artefacts linked to the space-for-time methodology that we adopt nor to the uncertainty in satellite retrievals. A first possible artefact is linked to the spatial granularity of the analysis, both in terms of the spatial resolution of the input CFrC data and the spatial window over which the space-for-time substitution is considered. By using an alternative implementation of the same data but with a higher spatial resolution, which is only available over Europe, we demonstrate that the patterns of change remain comparable when considering a finer scale (see ‘Methods’ section for details). A second possible artefact stems from the bias in the location of the forest within the landscape. In many areas around the world, forests occupy the more marginal lands that are less suitable for agriculture, such as humid zones, areas with shallow soils and/or sloped terrain^56^. However, when repeating the analysis using an alternative methodology^21^, which is based on the analysis of areas in which forest cover has effectively changed (instead of the potential change represented by neighbouring contrasting areas in the space-for-time method), results remain largely consistent in terms of sign, albeit with different magnitudes that can be explained by the difference in nature of the transition (see Fig. 3 and ‘Methods’ section for details). A final confirmation of our results comes from independent ground observations of CFrC recorded from SYNOP stations across Europe (see Fig. 4a and ‘Methods’ section for details). By confronting CFrC values from paired sites with differences in forest cover, a clear rise in CFrC is evident from April to August and from late morning until mid-afternoon (Fig. 4b). Despite a difference in magnitude, the seasonal excursion at the satellite overpass time (circa 14:00 local time) largely follows the seasonal pattern obtained from space over the corresponding area, with the notable exception of the negative values in February and March (Fig. 4c).

### Possible underlying physical mechanisms

It is beyond the scope of this study to disentangle and attribute all the physical processes acting towards generating or inhibiting cloud formations above forests across the world. These processes will vary considerably depending on various factors such as weather, climate, vegetation physiology and soil moisture. While in some places cloud occurrence could be limited by the heating needed for uplift, in some it could be limited by moisture supply. The relative importance of various different factors across different geographic locations would need dedicated studies based on modelling experiments where different factors can be isolated (e.g.^57^). Such studies could build up from our present work by using it as a benchmark that models could strive to reproduce, and by doing so contribute to the increased understanding of the underlying mechanisms. Having said that, we do provide here a brief discussion of possible drivers behind patterns we see in the resulting dataset.

Snow is a first driver that could affect the patterns in CFrC change that we observe. When land is covered by snow during winter and spring over northern latitudes, we see a clear reduction of CFrC above forests with respect to open lands. A plausible reason for this pattern lies in differences in air stability above forests and snow-covered open lands. In snow-covered conditions, the large sensible heat flux driven by the larger net radiation of forests combined with the low winter transpiration favours the development of a deep dry boundary layer that is more effective in the vertical transport of momentum as compared to open areas^1,58^. This dry boundary layer prevents the development of low-level clouds, while over the neighbouring snow-covered open lands the low value of net radiation leads to stable stratification and an increased probability of persistent fog. This radiation fog typically develops during the persistent anticyclonic conditions during the winter, such as the Siberian High in northern Eurasia^59^. Incidentally, fog is also the most probable reason why the negative change in CFrC is not detected by ground observations (Fig. 4), as these stations do not typically report CFrC when the sky is not visible in the presence of fog. Finally, confusion between clouds and snow can partially occur in the satellite CFrC product, which would artificially lead to more (erroneously) detected clouds above open lands due to their higher snow coverage.

Water availability is another factor that can alter the general pattern of cloud formation over forests. Forests would typically have more access to ground water than neighbouring grasses due to their deeper roots, thus naturally favouring more cloud formation. However, the case of the Indian subcontinent (Fig. 1j and Supplementary Fig. 3) illustrates how the magnitude of this effect can strongly change along the season depending on water availability. During the dry season from December to April, trees still have access to water resulting in a higher CFrC than neighbouring crops/grasses. In May, this difference drops dramatically as air temperature rise strongly and water becomes scarce for all plant types. Then, the changes in CFrC remain low during the monsoon period (June to August), when the coupling between the land and the atmosphere is expected to be weak, only to progressively return to higher levels as the rainy season recedes and water becomes increasingly less available especially for non-forested vegetation. In the beginning of the Monsoon, the CFrC even becomes slightly negative. One possible explanation might be that under a situation of no water stress for neither grasses nor trees, the former are bound to transpire more as they have a lower Bowen ratio (given their lower hydraulic and stomatal resistance), but further research would be needed to go beyond this speculation. In the US Corn Belt during summer we also observe a clear increase in cloud cover over croplands with respect to adjacent forests (Fig. 1g and Supplementary Fig. 3). This is in line with a recent study that shows how agricultural intensification in this area is a major contributor to the observed increase in summer rainfall^35^. A modelling study has similarly shown that C4 plants (such as corn) would have higher cloud cover than C3 plants under similar conditions due to their higher water efficiency which translates into more vigorous buoyant thermals^30^. Finally, we should also recognize that irrigation has a strong biophysical effect^60^, which should also translate in changes of cloud cover, but we could not explicitly isolate the effect of irrigation in our current experimental set-up (see ‘Methods’ for details).

Landscape heterogeneity has also been reported as a driver of cloud formation by both modelling^28,61,62^ and observational experiments^49^. This has notably been seen in the Amazon following deforestation, which typically starts along road networks following a characteristic fishbone pattern, which then evolves into a heterogeneous complex dominated by croplands or grasslands mixed with forest remnants and settlements. Such a fragmented landscape has a larger roughness length than the neighbouring intact and homogeneous forest, which enhances exchange of energy and momentum, generating a stronger lifting mechanism and thus a relatively more active shallow convection regime over deforested areas^49^. This effect has been clearly observed during the dry season (around August) and has repercussions with the amount of rainfall during that period^63^. The estimations we provide over the Amazon basin do follow this pattern of marked increase of CFrC over deforested areas (Fig. 1h and Supplementary Fig. 3).

**Fig. 5:**
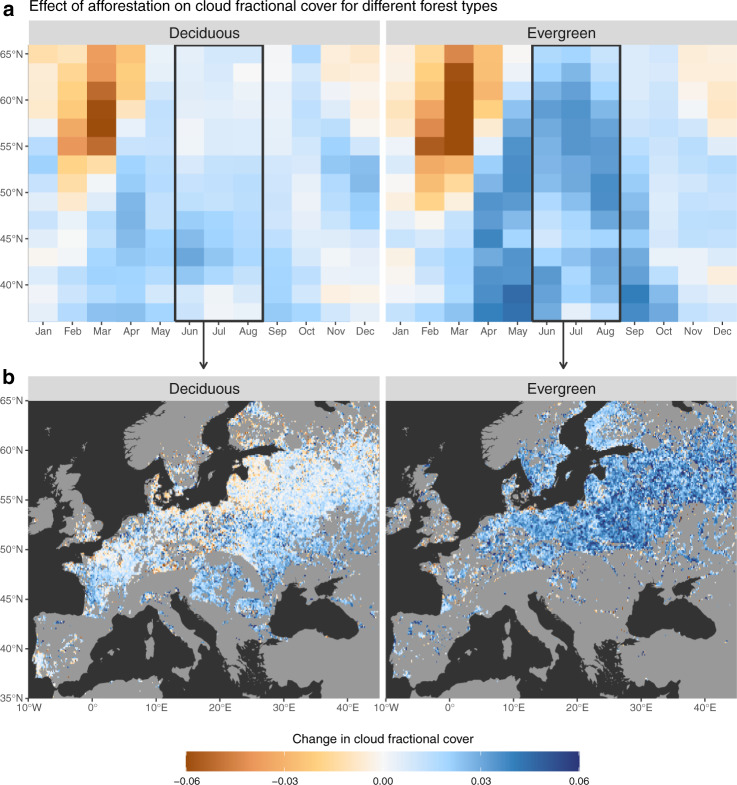
Change in cloud fractional cover following afforestation of two different forest types: deciduous forests or evergreen forests. **a** The latitudinal monthly averages over the entire areas. **b** The spatial distribution of the averaged summer values.

Finally, as mentioned before, another driver that could also affect the expected patterns of CFrC change are BVOCs. More specifically, we speculate that the high values of CFrC enhancement observed over Eastern Australia may be related to the predominance of Eucalypts trees, which typically emit large quantities of BVOCs. Another place where BVOCs would have a considerable role is in conifer forests^53^. This factor, combined with the higher Bowen ratio of needleleaf trees, may partly explain the fact that systematically higher values of CFrC are detected over needleleaf forests compared to deciduous ones (see Fig. 5).

### Implications for land-based mitigation

The widespread enhancement of cloud cover we observe over most forests consolidates the notion that land-based mitigation through afforestation, forest restoration or avoided deforestation should not be reasoned purely in terms of carbon sequestration^1,16^. Although our analysis does not directly relate cloud formation to rainfall, we can anticipate that the cloud formation we see could strengthen the hydrological value of forests. In a world projected to become regionally drier^64^, such increase in water availability from self-generated rainfall is an asset to make ecosystems more resilient, and thus more adapted to the changing climate. This may in turn become directly relevant for sustaining the terrestrial carbon sink, as the latter is strongly affected by inter-annual fluctuations in terrestrial water storage^65^ and its inter-annual variability is dominated by semi-arid ecosystems^66^. Clouds further increase the value of forests for climate mitigation by increasing planetary albedo. While metrics have been proposed to quantify the direct biophysical effect of lowering the surface albedo following afforestation^67^, these ignore the associated indirect effect of increased cloud cover. Given the complexity of the shortwave and longwave radiative budgets of clouds and the associated feedback onto other elements of the climate system, we are not capable of quantifying the actual changes in radiative forcing generated by changes in forest cover using observational data only. Modelling studies at sufficiently fine spatial and temporal resolution would be necessary to do so. However, by design our methodology is only sensitive to low clouds that have mostly cooling effects^40^, and it is reasonable to assume that forests would have at best a limited effect on the direct formation of high clouds with warming effects. Therefore, our results still suggest that the overall effect of afforestation through indirect cloud formation should be aligned with carbon sequestration to cool the Earth, even if this may certainly vary depending on location and tree type.

The need for a comprehensive view on the climate impacts of forests is particularly timely and relevant for Europe. A set of policy initiatives known as the European Green Deal have the overarching aim of making Europe climate neutral by 2050. Although the European Green Deal is currently stimulating afforestation, forest-based mitigation efforts in Europe have come under scrutiny from recent model-based studies^11,68^. According to the first assessment, afforestation and forest management in Europe during the past 250 years have not mitigated climate warming^68^, partly because these operations involved a transition from brighter broadleaf species to darker conifer forests. The second study^11^ argues that because of unavoidable trade-offs between climate objectives, such as sequestering carbon versus reducing air temperature thanks to albedo management, Europe should not rely on forest management for climate mitigation. Given the substantial uncertainty that models still have to characterize biophysical effects of land cover change, notably in terms of partitioning of latent and sensible heat fluxes^23,69^, plus the complexity in modelling cloud-forming processes at fine scale, the conclusions of these studies relying on the albedo change between forest types may have to be revisited in the light of the observation-driven analysis we provide here. To stimulate discussion on this topic, we further provide a tailored diagnostic separating broadleaf from evergreen forests at a finer spatial scale (Fig. 5). We show that the cloud generating effect is particularly stronger for the evergreen (consisting predominantly of conifers), probably due to a combination of larger roughness lengths, lower albedo (that increase net radiation) and lower stomatal conductance (that increase the Bowen ratio). All together, these functional properties of needleleaf forests are expected to increase the sensible heat flux, which could stimulate cloud formation despite the reduction in moisture through transpiration (with respect to deciduous forests). As a consequence, the warming effect from darker needleleaves may actually be counterbalanced by a higher increase in cloud albedo. This could add considerably more value than previously thought to the climate services provided by European forests, particularly for those based on dark conifers.

Snow over land changes the story. When snow is present, forests appear to cause a reduction in cloud cover, which leaves the forests more exposed to direct sunlight, thus amplifying their darkening effect and thereby reducing their overall mitigation potential. Further research is needed to quantitatively evaluate whether this positive forcing in winter counterbalances the negative forcing of higher cloud cover during summer. However, given the substantially higher radiation load in summer, it is unlikely. Furthermore, in a warming world the snow cover duration is projected to decrease^70^, and therefore the positive effects on cloud cover should increasingly outbalance the negative ones. Given the necessary time to establish new forests, the negative effect would therefore be less relevant for climate mitigation then than it is now.

### Summary and perspectives

This study provides a global comprehensive observation-driven assessment of how forests alter their overhead cloud regime at local level. The main message that emerges from our assessment is to underline the far-reaching effect of forests and land cover change on our climate. Our results suggest that the indirect climate effect of forests through changes in low cloud formation are generally aligned with carbon sequestration to cool the Earth. In other words, an effort to mitigate climate change through afforestation is likely accompanied with an additional cooling effect due to increased planetary albedo. Overall, this indirect biophysical effect would likely counteract, on average, the darkening of the surface following afforestation, adding further climatic value to forests beyond that of carbon sequestration and local surface cooling by evaporation. Furthermore, the increase in cloud cover could translate into more precipitation, giving forests additional hydrological value, and also potentially reducing the vulnerability of the carbon stocks, the occurrence of forest fires and strengthening their adaptation capacity in a changing climate. When considering the extra ecosystem services provided by forests, such as preventing soil degradation and enhancing biodiversity, our results consolidate the rationale for supporting actions favouring avoided deforestation, forest restoration and afforestation.

Our assessment is driven by observations, which provides solid empirical evidence to contribute to our global understanding of plant–climate interactions. However, this also comes at a price in terms of limits to how far we can deepen the analysis and attribution of the underlying mechanisms. For instance, due to the multitude of processes involved, it is hard to imagine how the cooling effects of land-based cloud formation could be robustly quantified in terms of radiative forcing at the top of the atmosphere from observations alone. It would be similarly hard to quantify the added value of potential afforestation in terms of precipitation without using some modelling framework. The observational nature of the study also limits how much we can disentangle the confounding drivers behind cloud formation (e.g. if clouds are formed because the forests are darker creating uplift, or because they transpire more, or both). Moreover, our method can only be expected to grasp a fraction of the local effects of land cover change on cloud cover, as processes such as advection are bound to reduce the signal we observe (see discussion in the ‘Methods’ section). To explore the full picture, including non-local effects, the only option is to combine experimental evidences with the formal representation of processes in Earth system model experiments.

We believe our observation-based assessment will serve as an invaluable benchmark that can serve to evaluate and constrain the models, possibly exploiting novel approaches involving deep learning to derive model-oriented knowledge from large datasets of Earth observations^71^, in order to improve model parametrization and reduce simulation uncertainties. This should enable a better calibration of the models and allow their use to derive an enhanced understanding of the mechanisms behind the consequences of changing the biophysical properties of the land.

## Methods

### Assumptions for the space-for-time substitution

The main methodological concept in this study is the notion of a space-for-time substitution. Such approach has previously been used in various studies to estimate the effect of land cover change on temperature^20,22^ or on the surface energy balance^22,72^. The overarching assumption behind the method is that the difference in properties of neighbouring patches of land can serve as a surrogate for changes in time. While this main assumption largely holds for land surface properties, such as skin temperature, it requires a more detailed articulation into several underlying assumptions in order to apply the approach to atmospheric properties such as cloud cover. This is because atmospheric properties are prone to lateral movements, partially decoupling them from the land cover directly below them, and thus adding considerable complexity to the analysis.

The first underlying assumption is that the method will be mostly sensitive to low-level convective clouds generated in the boundary layer (i.e. cumulus clouds). These are typically formed under stable conditions of high pressure and low wind, and are thus expected to have a higher spatial correlation with the underlying landscape elements. Other types of low-level clouds, such as stratus clouds, are typically much more uniformly spread across the landscape, which would result in no difference in CFrC when comparing two distinct and neighbouring vegetation classes. For medium- or high-level clouds, their position will be determined mostly by the state of the atmosphere rather than by the land surface, resulting in a very low correlation with vegetation spatial patterns. The space-for-time substitution approach would thus similarly result in white noise.

The second assumption is that the boundary layer cumulus clouds will see very limited lateral advection between the moment of their formation and the satellite observation. Cumulus clouds over land show a very stable climatology where the peak formation is largely confined to the early afternoon (around 14:00), timing which remains very stable across space and season^43^. Therefore this assumption should largely hold if the observations are made at this time.

The third assumption is that if we consider topographically flat terrain that is away from a coastline, general weather conditions are essentially the same at a local scale (i.e. a region of radius circa 25 km around a given point). Within such an area, we then assume that variations in low cloud cover are mostly determined by local differences in surface properties, themselves determined by the type and condition of the present land cover.

### Preparation of input datasets

This study requires gridded geospatial datasets for two variables: cloud fractional cover and land fractional cover. Both datasets used here have been prepared in the frame of the European Space Agency’s (ESA) Climate Change Initiative (CCI)^73^.

The Cloud CCI^55^ provides a series of cloud properties derived from distinct satellite Earth observation platforms in a harmonized way. Here we use their cloud fractional cover variable (henceforth CFrC), which describes the fraction of a 0.05° × 0.05° pixel covered by clouds based on observations made at a finer spatial resolution at the given time of the satellite overpass. We chose to use Cloud CCI dataset based on the MODIS instrument on-board of the Aqua platform for two reasons. First, the timing of overpass of the Aqua platform (circa 13:30 local time at the Equator) coincides very well with the timing of peak of cumulus cloud formation^43^, thus greatly limiting the extent of possible cloud advection between the moment of cloud formation and observation. Second, native spatial resolution of the MODIS instrument is superior to the alternative (AVHRR), and should result in a better sensitivity to the presence of small cumulus clouds. More specifically, out of the 5 spectral bands of the MODIS instrument used by the Cloud CCI to characterize cloud properties (bands 1, 2, 20, 31 and 32), two of them (bands 1 and 2) have a native spatial resolution of 250 m. While these are aggregated to 1 km (the spatial resolution of the other MODIS bands) prior to their ingestion in the cloud retrieval algorithm, their finer native granularity and quality should prove to be an asset for small cumulus cloud detection. The CCI MODIS-AQUA CFrC data is available for the period 2004–2014. The values are first averaged from daily to monthly scale, and then a single monthly value is calculated for every pixel over the period 2004–2014. The results are 12 layers each representing the multi-annual average CFrC for a given month.

The second type of data needed for the analysis is the fraction of the 0.05° × 0.05° pixels that are covered by distinct vegetation types (essentially trees and grasses) and by other land cover classes (urban areas, bare soil, etc.). These are derived from the Land Cover CCI^54^, a set of consistent annual maps describing, with a spatial resolution of 300 m, how the terrestrial surface is covered based on The United Nations Land Cover Classification Scheme^74^. This information is aggregated both spatially and thematically using a specifically designed framework^75^ to produce maps of general land fractional cover with a spatial resolution of 0.05° to match that of the cloud fractional cover data. The procedure is very similar to that done in a previous study^22^. For the context of this study, which has a focus on afforestation, the interest lies on transitions among three main vegetated classes, namely: deciduous forest, evergreen forests and herbaceous vegetation. Herbaceous vegetation is composed of both grasses and crops, irrespective of management practice such as irrigation. While irrigation has a clear biophysical effect of its own^60^, we deemed the land cover product was not consistent enough for this specific class. For reasons that are explained in the respective methodological section below, the full compositional description of the landscape is necessary (i.e. beyond the classes of interest), and therefore land cover fractions of the following classes are also generated: shrublands, savannas, wetlands, water, bare or sparsely vegetated, snow or ice, and urban.

### Retrieving potential cloud fractional cover change

Under the above-mentioned assumptions, we apply a space-for-time substitution algorithm developed in a previous study^22^ to the cloud fractional cover and land fractional cover datasets. We summarize the main aspects of the methodology, along with the few necessary adaptations, but the reader requiring more detail is redirected to the original papers^22,76^. The approach consists in applying an un-mixing operation over a spatially moving window containing *n* pixels. Over each window we apply a linear regression based on a matrix **X** containing the explanatory variables, in which each column of **X** represents the fractional cover of a given land cover type for each of the *n* pixels. The response variable is a vector **y** containing the *n* values of CFrC for the *n* pixels, while the vector **β** represents the regression coefficients:

1 $${\bf{y}}={\bf{X}}\beta$$

This is equivalent to solving the following system of equations:

2 $$\left\{\begin{array}{ll}{y}_{1}=&{\beta }_{1}{x}_{11}+{\beta }_{2}{x}_{12}+...+{\beta }_{m}{x}_{1m}\\ {y}_{2}=&{\beta }_{1}{x}_{21}+{\beta }_{2}{x}_{22}+...+{\beta }_{m}{x}_{2m}\\ \vdots &\\ {y}_{n}=&{\beta }_{1}{x}_{n1}+{\beta }_{2}{x}_{n2}+...+{\beta }_{m}{x}_{nm}\end{array}\right.$$

in which the digits of the subscript of *x*, e.g. *x*_*ij*_, represent the land cover fraction *j* in pixel *i*, for the *n* pixels in the moving window and the *m* classes that are considered. Once identified, we can use the *β* coefficients to predict the local *y* value corresponding to a given composition *x*, including that composed of a single land cover *j* by setting *x*_*j*_ = 1 and all other *x* values to zero. However, applying a regression directly on **X** carries a risk due to the compositional nature of the data (i.e. the sum of each row adds up to one), as the analysis of any given subset of compositional components can lead to very different patterns, results and conclusions^77^. To avoid this, we reduce the dimensionality of **X** through singular value decomposition (SVD) after removing the mean of each column:

3 $$({\bf{X}}-{\bf{M}})={\bf{U}}{\bf{D}}{{\bf{V}}}^{t}$$

where **M** is the appropriate matrix of column means, **U** and **V** are the matrices containing, respectively, the left-hand and right-hand singular vectors, and **D** is a diagonal matrix containing the singular values representing the standard deviations of the ensuing dimensions. The squared values of **D** represent the variance explained by each dimension, and can thus serve to define *z*, a reduced subset of dimensions that conserves 100% of the original matrix’s variation. The corresponding *z* right-hand singular vectors, **V**_*z*_, can then be used to find the appropriately transformed predictor matrix of reduced dimension **Z** as follows:

4 $${\bf{Z}}=({\bf{X}}-{\bf{M}}){{\bf{V}}}_{z}$$

which can now be regressed onto the CFrC *y*:

5 $$y={\bf{Z}}{\beta }_{z}+\varepsilon$$

where **Z** has been augmented with a leading column of 1s to accommodate an intercept term in the regression. We then use the standard method to obtain an estimate of *β*_*z*_:

6 $${\beta }_{z}={\left({{\bf{Z}}}^{t}{\bf{Z}}\right)}^{-1}{{\bf{Z}}}^{t}y$$

However, because of the matrix transformation from **X** to **Z**, the regression coefficients *β*_*z*_ do not provide direct information on the relationship between land fractional cover and cloud fractional cover (as in a normal regression). To identify the *z* values associated with a particular vegetation or land cover type (within the local analysis defined by the moving window), we define a ‘dummy pixel’ whose composition contains only a single class, with all other classes in its composition set to zero. This pixel’s composition is then transformed, and its *y* value predicted. This is the *y* associated with that vegetation type. To generalize this for all compositional components of interest, we define a matrix **P** with as many rows as these compositional components that we wish to predict. **P** is centred on the same column means as above (**M**, specific to each local analysis), and then multiplied by the correct number of transposed right-hand singular vectors (**V**_*z*_, again, specific to each local analysis).

7 $${{\bf{Z}}}_{{\rm{p}}}=({\bf{P}}-{\bf{M}}){{\bf{V}}}_{z}$$

Predicted *y*_p_ values for each vegetation or land cover type (identified by predicting the appropriately transformed ‘dummy pixels’) are then calculated as:

8 $${y}_{{\rm{p}}}={{\bf{Z}}}_{{\rm{p}}}{\beta }_{z}$$

The expected change in variable *y* associated with a transition from vegetation type A (e.g. herbaceous vegetation) to vegetation type B (e.g. deciduous forest) at the centre of the local window is then the difference between the *y*_p_ predicted for each ‘pure’ vegetation type:

9 $${{\Delta }}{y}_{{\rm{A}}\to {\rm{B}}}={y}_{\rm{B}}-{y}_{\rm{A}}$$

The uncertainty in the estimation of Δ*y*_*A*→*B*_ can be expressed as a standard deviation using the following expression:

10 $${\sigma }_{{\rm{A}}\to {\rm{B}}}=\sqrt{{\sigma }_{\rm{A}}^{2}+{\sigma }_{\rm{B}}^{2}-2{\sigma }_{\rm{AB}}}$$

where ${\sigma }_{\rm{A}}^{2}$ and ${\sigma }_{\rm{B}}^{2}$ are the variances in the estimates of *y*_A_ and *y*_B_, and *σ*_AB_ is their covariance. These variances and covariances are in turn obtained from the covariance matrix, defined from the regression as:

11 $${\mathbf{\Sigma }}={{\bf{Z}}}_{{\rm{p}}}{\rm{Var}}[\beta ]{{\bf{Z}}}_{{\rm{p}}}^{t}$$

The diagonal terms in **Σ** are the variances of individual predictions of (individual) classes. The off-diagonal parts of **Σ** hold the covariances between these predictions. As a reminder, the uncertainty *σ*_A→B_ calculated in this way is related to the methodological uncertainty and does not include the uncertainty in the input variables of land cover or cloud fractional cover.

In the default set-up for this study, we concentrate on two transitions: herbaceous vegetation to deciduous forest and herbaceous vegetation to evergreen forest. These are calculated using a spatial window of 7 × 7 pixels, each pixel being of 0.05°, resulting in a squared spatial window of circa 35 km in size. To ensure there are enough values to do the un-mixing over each window, we established that there must be a minimum of 60% of valid values in each window, and that at least 40% must have distinct compositions. The operation is applied to all 12 monthly layers of CFrC, resulting in 12 maps of Δ*y* with a 0.05° spatial resolution for each of the two vegetation cover transitions.

### Post-processing

A series of post-processing steps are required to ensure the results of the Δ*y* maps can be used to evaluate the effect of land on cloud cover. The first step is to mask all pixels in which there is insufficient co-occurrence of the two vegetation classes involved in the transition. This co-occurrence is quantified by an index of vegetation co-occurrence^76^, *I*_c_, calculated from the land fractional cover layers using the same spatial moving window of 7 × 7 pixels as used before. This index is calculated pairwise, i.e. for 2 vegetation classes of interest A and B, using two vectors *p*_*A*_ and *p*_*B*_, describing the presence of these two vegetation classes in each of the *i* pixels in the moving window. It also requires the definition of another *i* point evenly distributed along a hypothetical line *B* = 1 − *A* in the two-dimensional space describing the presences of vegetation class A and vegetation class B. These points, whose position in the 2-D space are labelled *q*_*A*_ and *q*_*B*_, represent an ideal situation of maximum co-occurrence that serves as a reference to establish the index. The formal definition of the index is thus:

12 $${I}_{{\rm{c}}}=1-\frac{{\sum }_{i}\min \{\sqrt{{\left({q}_{A}-{p}_{A}\right)}^{2}+{\left({q}_{B}-{p}_{B}\right)}^{2}}\}}{{\sum }_{i}\sqrt{{q}_{A}^{2}+{q}_{B}^{2}}}$$

The minimum operator in the numerator selects the smallest distance that a given point *p* can have to any of the *q* points. The sum relates to the sum of this distance for all *i* points in the spatial moving window. The denominator characterizes the maximum distance that the point *p* can encounter. *I*_c_ will range from 0 to 1 corresponding to a gradient of ‘no presence of either class’ to ‘full and evenly balanced presence of both classes’. As in^76^, we retain only pixels with *I*_c_ ≥ 0.5 where we consider that there is sufficient information at local scale concerning both vegetation types to derive meaningful information about the target land cover transition.

The second step is to remove the potential orographical effects, which can be especially problematic given that forests are more likely to be located over mountainous areas due to human action^56^. Here we mask the areas where considerable topographical variation occurs within the 7 × 7 pixel moving window of interest using the same implementation described in^76^. This involves using 3 different indicators, *v*_1_, *v*_2_ and *v*_3_, calculated over the moving window based on *μ*_*h*_ and *σ*_*h*_, which are, respectively, the mean and the standard deviation of elevation over each grid cell of the input cloud dataset. These are defined as follows:

13 $${v}_{1}=\frac{1}{n}\mathop{\sum }\limits_{i=1}^{n}{\sigma }_{h,i}$$

14 $${v}_{2}=| {\mu }_{h}-\frac{1}{n}\mathop{\sum }\limits_{i=1}^{n}{\mu }_{h,i}|$$

15 $${v}_{3}=| {\sigma }_{h}-{v}_{1}|$$

For an interpretation of these metrics, readers are invited to read^76^. These three indicators are combined together in a single layer depicting all pixels satisfying all of the following conditions: *v*_1_ < 50 m, *v*_2_ < 100 m and *v*_3_ < 100 m. Pixels that fail any of these conditions are masked out from all the layers of results.

The third step is to aggregate the information to a coarser spatial support corresponding to the moving window. This step combines a decorrelation operation with a weighted averaging to result in a single value for a new pixel with a spatial resolution of 0.35°. The decorrelation is necessary as every spatial moving window overlaps, resulting in highly auto-correlated results. The degree of auto-correlation is characterized by an *n* × *n* matrix **R**_*a*_ containing the fraction of overlap between every pair of windows. By combining **R**_*a*_ with **D**_*a*_, a diagonal matrix containing the estimation uncertainties for the central pixel of each window in its diagonal, we can build a new covariance matrix **Σ**_*a*_ (the subscript ‘a’ is used to differentiate these matrices involved in this aggregation step from those used before):

16 $${{\mathbf{\Sigma }}}_{{{a}}}={{\bf{D}}}_{{{a}}}{{\bf{R}}}_{{{a}}}{{\bf{D}}}_{{{a}}}^{t}$$

The vector of weights for the spatial aggregation from 0.05° to 0.35° that includes the decorrelation is then obtained as:

17 $${\bf{w}}=\frac{1}{{{\bf{1}}}^{t}{{\mathbf{\Sigma }}}_{{{a}}}^{-1}{\bf{1}}}{{\mathbf{\Sigma }}}_{{{a}}}^{-1}{\bf{1}}$$

which can then be used to calculate the aggregated $\overline{{{\Delta }}y}$ as:

18 $$\overline{{{\Delta }}y}=\mathop{\sum}\limits_{i}{w}_{i}{{\Delta }}{y}_{i}$$

while the aggregated uncertainty ${\sigma }_{\overline{{{\Delta }}y}}^{2}$ is given by:

19 $${\sigma }_{\overline{{{\Delta }}y}}^{2}={{\bf{w}}}^{t}{{\mathbf{\Sigma }}}_{{{a}}}{\bf{w}}=\frac{1}{{{\bf{1}}}^{{\bf{t}}}{{\mathbf{\Sigma }}}_{{{a}}}^{-1}{\bf{1}}}$$

Again, for more details on these operations, see^22,76^.

The methodological procedure is still susceptible to the generation of some unrealistic data. A reason for this might be uncertainties and errors in the input data, which could not be explicitly taken into account in the methodology. To mitigate their effect, we decided to filter for outliers. We first removed the aggregated $\overline{{{\Delta }}y}$ values that are calculated based on less than 20% of the underlying 0.05° pixel values. We also removed values in which the aggregated methodological uncertainty (${\sigma }_{\overline{{{\Delta }}y}}^{2}$) is above 0.1. We then removed data falling outside the 1st and 99th percentiles of the entire dataset for each transition.

The final post-processing step is to merge together the two separate datasets corresponding to the two different forest types (deciduous and evergreen). This is done using weighted averages based on their respective presence in each grid cell. A simplified version is also produced at a reduced resolution of 1°, useful for visualization purposes (e.g. in Fig. 1).

### Considerations on the space-for-time method

The indirect biophysical effects of land cover change discussed in this paper refer exclusively to local cloud cover (so-called ‘local effect’). Our methodology relies on spatial gradients in a local moving window, and it is therefore intrinsically incapable of quantifying large-scale non-local effects. However, it has been shown with modelling studies that changes in forest cover can also have relevant non-local biophysical effects. For instance, albedo-induced cooling following deforestation is mainly a non-local effect through mechanisms of teleconnections that may ultimately compensate the opposite local warming effect^78^. It is unclear how potential non-local effects would affect the local effects we observe. Modelling experiments with dedicated coupled runs would need to be established to investigate this further, but they would require a fine granularity to adequately represent the local changes in land cover.

A remark is warranted to discuss particular situations where there might be a strong influence of water on the space-for-time substitution signal. For example, trees may be expected to behave differently if planted over wetlands. We deemed that cases involving afforestation in wetland would deserve a specific attention that is beyond the scope of this more general global analysis. For this reason, we kept wetlands as a separate vegetation cover class, whose effect is therefore factored out when considering the transitions of afforestation from herbaceous vegetation to either evergreen forests or deciduous forests. Irrigation, on the other hand, is included in the herbaceous vegetation class. Therefore, if we consider potential afforestation in areas where irrigated crops are presently common, the resulting estimated CFrC change will relate to that of a transition from irrigated cropland to forest. Finally, another place where water could affect the method includes coastlines. Their effect are mitigated by the experimental set-up of the moving window, which requires that more than 60% of the pixels be valid non-water pixels. This generates a buffer around coastlines for which the values are ignored, precisely to avoid these areas where the cloud formation may be dominated by the sea. Local residual coastline effects might still remain is some places, and further methodological refinements could be foreseen to where the method be applied for a regional scope.

Despite having observations coincide with the peak formation of cumulus clouds, clouds might still move. Several methodological points should mitigate the consequences of this effect on our results. First, we work on monthly averaged CFrC, which is then averaged over the 11-year period to describe a multi-annual cloud regime for every grid cell irrespective of all wind conditions (in terms of both strength and direction) encountered during this period. In the unlikely event that at some places the wind climatology has indeed a systematic direction and a sufficient strength to displace the low-level boundary clouds far beyond the underlying forest in the short amount of time they have, the most likely result is a lack of correlation between the land and the atmosphere. This would lead to the same result as what we described in the assumptions for stratus or high clouds: the space-for-time method would lead to random noise. Cloud movements can actually be expected to effectively decrease the spatial gradients (among different neighbouring land cover classes) that we are studying, since these would be stronger in the absence of cloud movements. As a result, we expect that our methodology underestimates the actual magnitude of the changes in CFrC.

**Fig. 6:**
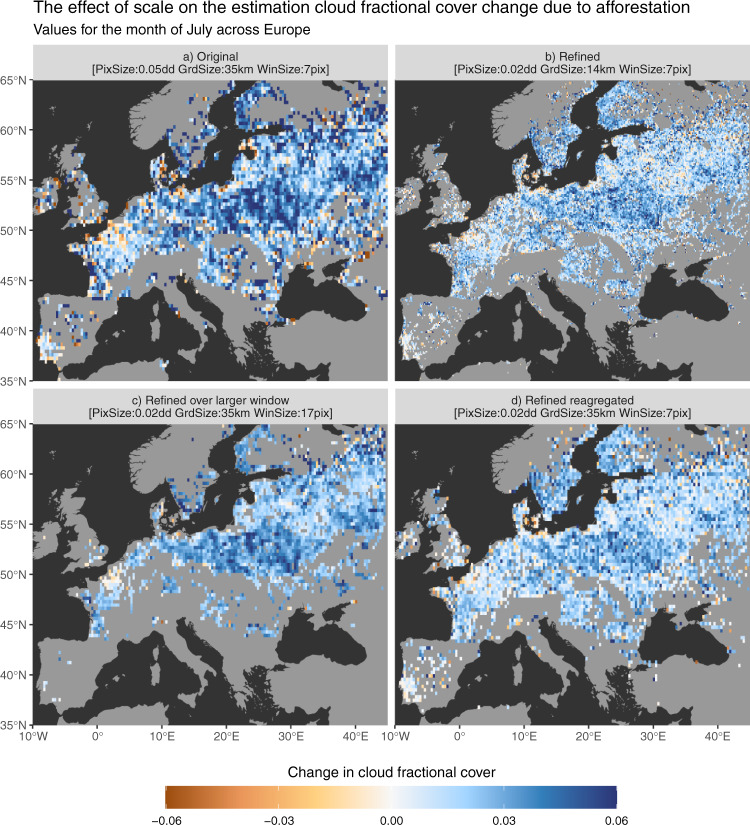
Exploring the effect of a change in scale on the methodology to extract the change in cloud fractional cover (CFrC) following potential afforestation over Europe. The original procedure applied at global scale with 0.05° data is presented in (**a**), while the same approach applied to a finer dataset of 0.02° is represented in (**b**). Panel (**c**) provides an alternative approach in which the fine spatial-scale data is used using a moving window of equivalent size to that used in the original method, thus involving more pixels. For comparison purposes, panel (**d**) provides the same information as panel (**b**), but aggregated to the same grid as those in (**a**) and (**c**).

Linked to the previous point, a special consideration is needed to the question of scale. The fact that convective clouds are susceptible to move laterally between the moment they are formed above the forest and the moment they are detected by the satellite also means that choosing the right spatial resolution to do the analysis is not directly evident. The finer the pixel, the more likely it is that the clouds have moved to neighbouring pixels. On the other hand, if the pixel is too coarse it may dilute the overall effect, leading further to underestimation of the magnitude. To partly explore the sensitivity of the methodology to scale, we realized some variant experiments. Over Europe, the Cloud CCI project additionally provides the same MODIS-based data product with a finer spatial resolution of 0.02° (instead of the standard 0.05° resolution). Using this specific dataset, two experiment variants have been made. The first consists of applying the space-for-time methodology with the same configuration: i.e. a moving window of 7 × 7 pixels, but here resulting in a finer scale with aggregated pixels of circa 14 km instead of circa 35 km. The second instead considers a 17 × 17 pixel moving window covering an area of circa 35 km, similar to the original experiment using 0.05° data. To do both of these specialized experiments, the land fractional covers had to be generated again from the CCI Land cover data to result in land fractional cover layers at the corresponding spatial resolution of 0.02°. The results of this analysis are summarized in Fig. 6, where it appears clear that the general patterns remain constant, but that the magnitude of the effect can depend on scale.

### Confrontation with an alternative method

In order to strengthen confidence in our results based on the space-for-time substitution approach, we also explore an alternative method. This other approach investigates the relationship between land cover change and biophysical effects by quantifying their effects after real land cover changes occur^21^. This method requires factoring out the local effect from climate variability to isolate the effect from the land cover using a similar moving window approach. For this purpose, the difference in monthly CFrC (Δ*y*) between two years at a given location is expressed as the effect of forest cover change (Δ*y*_fcc_) plus the residual signal (Δ*y*_res_) due to climate variability, so the former can be estimated as such:

20 $${{\Delta }}{y}_{{{fcc}}}={{\Delta }}y-{{\Delta }}{y}_{{{res}}}$$

To increase the number of samples that can be used with this approach, the calculation is done based on all possible pairwise comparisons of different years within the 2004–2014 period, not just between the first and last year. This is applied independently for each month. We here use the same implementation as a previous study^21^, but we use a forest cover percentage derived from the CCI Land Cover to remain consistent with the space-for-time approach.

While these results would arguably be a more direct estimation of cloud cover, there are two disadvantages that preclude it from using it as the main approach. First, the number of actual changes in land cover is much smaller and harder to identify than the potential changes used in the space-for-time approach, resulting in a strongly reduced number of observations from which to derive an estimation (and thus a necessity to have larger and less reliably stable moving windows). Second, land cover change is a gradual process and thus the effect of the change (e.g. change in cloud cover regime in this case) may not be fully expressed in the temporal interval considered. These reasons explain why the results are not expected to be identical to those obtained from using the space-for-time substitution. Their role here is to add confidence to the main analysis by showing that two different approaches do generally show the same direction in the response of cloud cover, as shown in Fig. 3.

### Confrontation with ground observations

To consolidate our CFrC change estimations from satellite we reproduce comparable estimations from ground-based observations of cloud cover from synoptic weather stations. These observations are made by visual examination of the sky under a specific protocol at regular sub-daily timesteps^79^. The global SYNOP database originated from the archive of the European Centre for Medium-Range Weather Forecasts (ECMWF) and assembles a harmonized set of such measurements across the world. However, the density and completeness of records is very variable geographically and temporally, with many more usable records in Europe than in the rest of the world.

To be usable for our purposes, we first need to associate a corresponding forest cover value to each station and filter out those for which cloud formation may be dominated by other factors. We assume here that the CFrC measurement at every SYNOP location relates to the fraction of forest cover within a region of influence defined by a radius of 15 km around each station. We thus associate to each point a value of forest cover fraction based on the Global Forest Change maps^80^ using the Google Earth Engine platform^81^. We prefer to use these simpler binary maps since their finer spatial resolution (30 m) is more appropriate for this fine-scale station-level analysis than the thematically richer CCI Land cover maps used for the space-for-time analysis. We similarly attribute to each station a value of water fractional cover related to the same 15 km radius using the global surface water maps^82^. This information is then used to filter out stations with more than 5% of water fractional cover where the micro-climate conditions may be strongly impacted by the presences of water bodies. A second filtering is based on the topography, using the same criteria as the one for the space-for-time analysis above. Finally, we only retain stations that have a minimum number of records corresponding approximately to 7 years of CFrC records between 2004 and 2014 in order to ensure we are not focusing on the situation of a single year.

The next step is to group stations in pairs that are comparable but which relate to variable percentages of forest cover. The main criteria we use to find such pairs is the distances between stations. A minimum distance is set to 30 km to ensure that there is no overlap between the regions of influences of the two stations, assuming that this equally prevents auto-correlation in terms of the sky viewed at each point. A maximum distance of only 100 km is tolerated to avoid the fact that the large-scale weather situation differs considerably in terms of the impact on cloudiness above the two locations. With these constraints, the resulting pair sites are only found in Europe where the network is dense enough (see Fig. 4a). The difference in CFrC Δ*y*_*synop*_ between both stations within each pair is computed, along with the differences in forest cover fraction Δ*x*_*synop*_.

The final step is to make the Δ*y*_synop_ compatible with the Δ*y* from satellite. The challenge is that the satellite estimation Δ*y*_A→B_ refers to a full transition from a land cover class to another (i.e. 100% cover of A to 100% of B), while there are no single pair of SYNOP points showing a transition of more than 40% of forest cover. To harmonize the two concepts, we extrapolate the theoretical value of a full cover transition from no forest to full forest by fitting a linear regression between Δ*y*_*synop*_ and Δ*x*_*synop*_ using the values from all pairs of points across Europe. As we force the relationship to pass through the origin, the slope represents the expected change in cloud cover for a full transition from no forest to forest. This regression is applied separately for every hour and for every month (resulting in Fig. 4b). To compare with the satellite estimates, the SYNOP values corresponding to 14:00 (close to the overpass time of the Aqua satellite) are compared to the total average of the satellite estimates falling within 50 km of the centroid of each station pair. This is done both with the original satellite data at 0.05° spatial resolution and the dataset with a refined 0.02° spatial resolution, both of which are summarized in Fig. 4c.

### Changes in the surface energy balance

We propose a final analysis confronting the potential changes in CFrC with equivalent changes in different components of the surface energy balance following potential afforestation. This is possible by leveraging on a previous study^22^, where the same space-for-time framework was used to quantify the changes in net radiation (*R*_*n*_), in latent heat flux (*LE*) and in the combination of sensible and ground heat fluxes (*H* + *G*). These datasets are fully described in^76^, in which two different levels of vegetation transitions are provided. The single transition between the class ‘forest’ and the class ‘crops and grasses’ of the more generic dataset (IGBPgen) is used here. We retain only the values where there are common spatio-temporal records of both datasets, i.e. those for changes in components of the surface energy balance (*LE*, *R*_*n*_ and *H* + *G*) and for changes in CFrC. By looking at how changes in these variables associated with afforestation co-vary with our estimated changes in CFrC, we can catch some insights on the underlying processes behind cloud formation above forests.

**Fig. 7:**
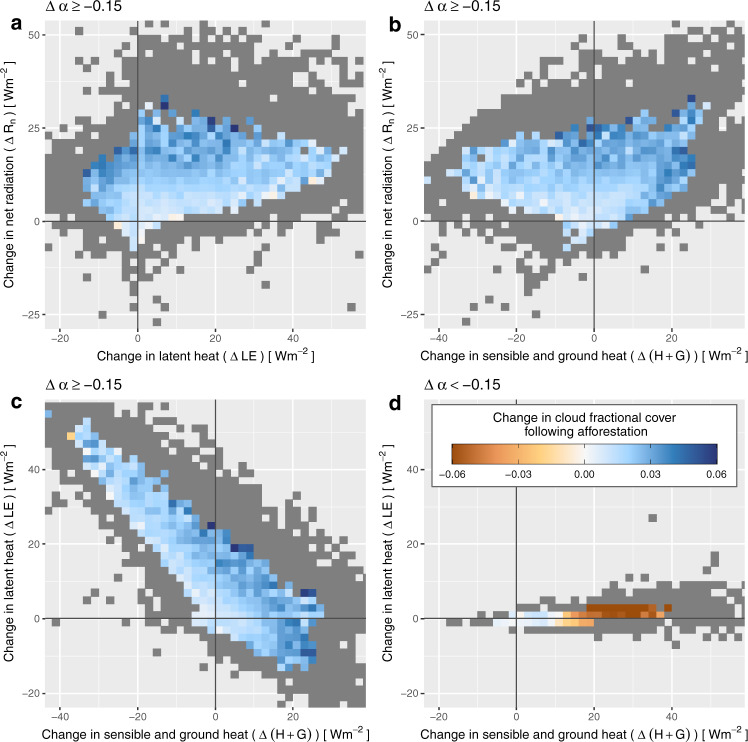
Displaying the change in cloud fractional cover (CFrC) following potential afforestation with respect to associated estimated changes in components of the surface energy balance. All pixels at all seasons are combined together and each grid cell represents an average of underlying points. Grey cells contain less than 15 records and thus are considered non-representative. The dataset is separated between records unaffected by snow in (**a**), (**b**) and (**c**) (where Δ*α* ≥ −0.15), and records in which there is a strong change in surface albedo due to snow (Δ*α* < −0.15) in (**d**). All data except for the changes in CFrC come from^76^.

This analysis brings all estimates of CFrC change in a common system of physical coordinates irrespective of their geographical location or their seasonal behaviour (Fig. 7). To facilitate the interpretation, we exclude snow-covered areas, by using a threshold on the reduction in surface albedo following afforestation (Δ*α* ≥ −0.1; such large differences in surface albedo occurs predominantly between dark emerging trees and snow-covered open lands). Once the snow effect is masked out, the general picture shows a clear increase in CFrC, with higher values when the afforestation entails a larger amount of net radiation (see gradients in Fig. 7a, b). When mapping the changes in CFrC with respect to changes in the non-radiative fluxes (Fig. 7c), we see that the large majority of cases where afforestation would occur (i.e. the points shown in the plot), there is either an increase in LE, in *H* + *G* or in both, and it is systematically associated to an increase in CFrC. This comforts the notion that cloud formation above forests are correlated to both the injection of extra moisture in the boundary layer and the increased bulk displacement of air generated by the fact the forest surface is darker. It also suggests that depending on where the potential afforestation occurs, one process might dominate over the other.

There are some shortcomings to this analysis that prevent us from drawing strong conclusions from it. First, unlike the CFrC product, the energy balance products are not specific to the early afternoon but instead resume the situation integrated over the entire day. This mismatch in the timing complicates the interpretation of a causal link between changes in surface energy balance and cloud formation, as the former are affected by other processes throughout the day (and night). Second, the original study^22^ assumed that the local indirect biophysical effects of vegetation cover change, such as the change in cloud cover we observe here, could be neglected in order to close the surface energy balance. Therefore, there is an inconsistency in this analysis, since the consequences on the energy balance of the change in CFrC are not reflected in the coordinates of the plots in Fig. 7. Despite these issues, we still think it is valuable to provide this analysis for completeness as a complementary description of the CFrC signal we have derived.

## Supplementary information

Supplementary Information

Peer Review File

## Data Availability

The datasets generated during the current study are available in the following Zenodo repository^83^: 10.5281/zenodo.4727774. This repository also includes all the source data necessary to reproduce all the figures in the present document. Maps have been made with vector files from https://www.naturalearthdata.com/.

## Code availability

The code used in this study, including the code to reproduce all the figures in the present document, can be found in the following Zenodo repository^84^: 10.5281/zenodo.4727822
