# Supplementary material for: Revealing the widespread potential of forests to increase low level cloud cover
Source: Nat Commun. 2021 Jul 15;12:4337. doi: 10.1038/s41467-021-24551-5 (PMC8282670; doi:10.1038/s41467-021-24551-5)
Supplement: Supplementary file 1 — Supplementary Information [file 41467_2021_24551_MOESM1_ESM.pdf]

Widespread cloud enhancement adds further value to the world's forests

June 8, 2021

Supplementary Information

Supplementary Figures 1 to 3

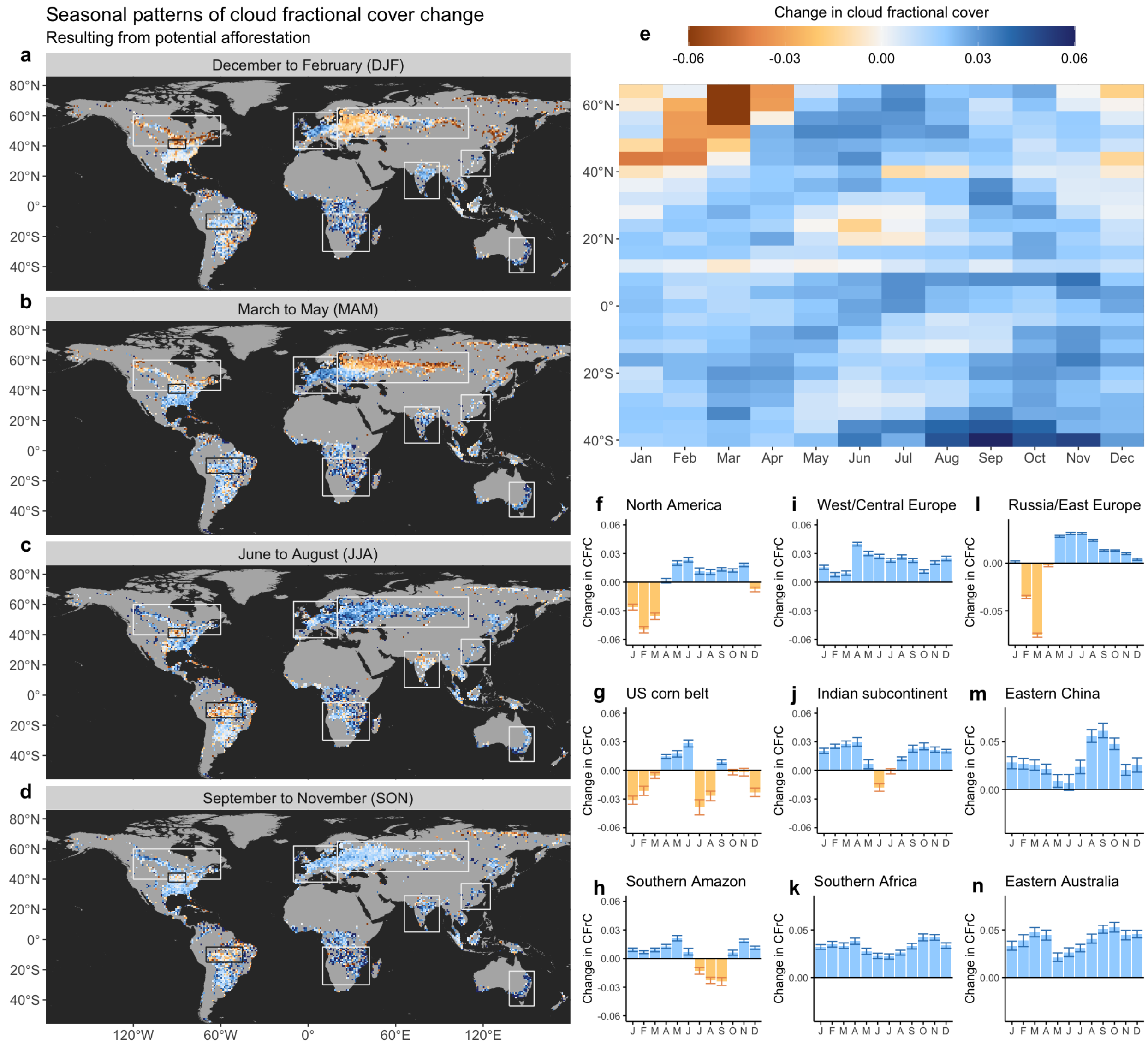

Supplementary Fig. 1 Changes in cloud fractional cover (CFrC) following potential afforestation as derived from satellite data. **a-d** Maps of seasonal patterns (aggregated to a reduced spatial resolution of 1 for visualisation purposes). **e** Latitudinal averages. **f-n** Seasonal behaviour of the mean change in CFrC over selected regions, each bar representing a month from January until December. The error bars represent the standard error around the mean. The grey areas in the maps represent places were no estimations are possible due to insufficient coverage of either forests or low vegetation. This is the same figure as Fig. 1 in the main text but representing cloud fractional cover (CFrC) in absolute instead of relative terms.

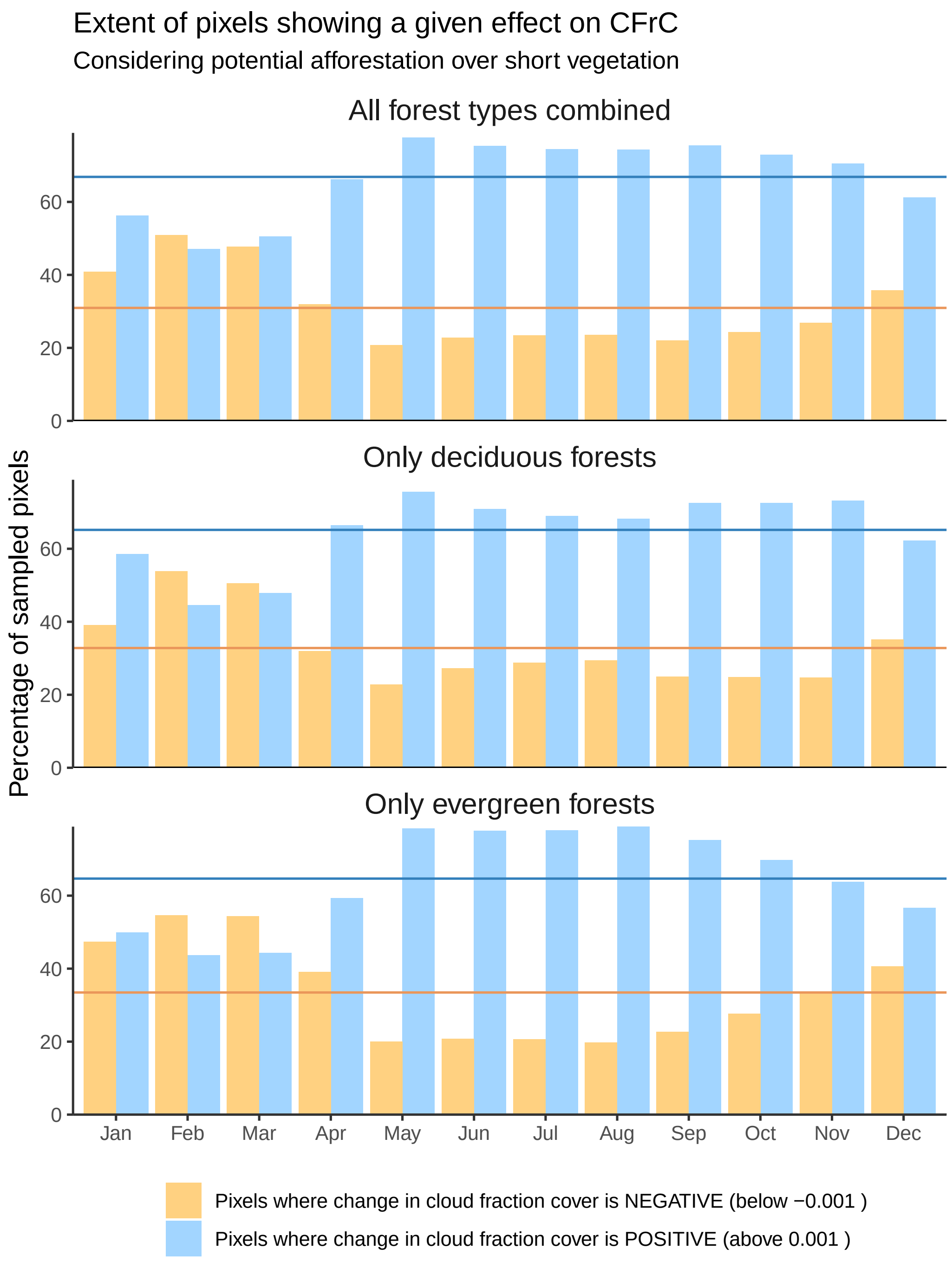

Supplementary Fig. 2 Seasonal patterns of the percentage of sampled pixels where forest has a positive (blue) or negative (yellow) influence on the cloud fractional cover (CFrC). For the southern hemisphere, the months have been cycled by 6 months. The horizontal lines represent the annual average. This is the same as Figure 2 in the main manuscript but here the separation by forest types is provided.

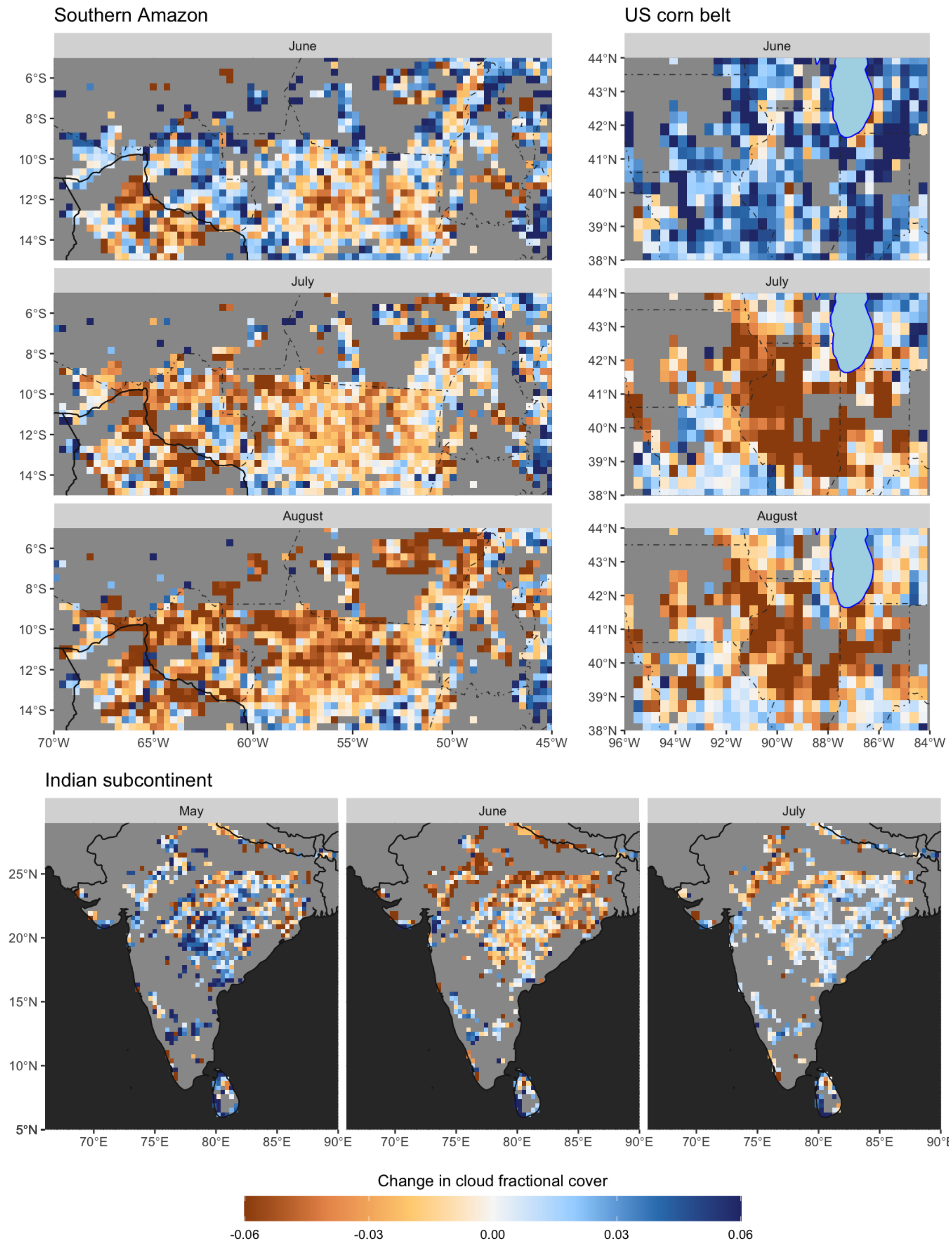

Supplementary Fig. 3 Detailed monthly maps of changes in cloud fraction cover (CFrC) for some of the regions highlighted in Fig. 1 in the main text and in Supplementary Fig. 1. Data are at the aggregated spatial resolution of 0.35 following the de-correlation operation.
