# Supplementary material for: Revealing the widespread potential of forests to increase low level cloud cover
Source: Nat Commun. 2021 Jul 15;12:4337. doi: 10.1038/s41467-021-24551-5 (PMC8282670; doi:10.1038/s41467-021-24551-5)
Supplement: Supplementary file 2 — Peer Review File [file 41467_2021_24551_MOESM2_ESM.pdf]

Peer Review File

Reviewer comments, first round –

Reviewer #1 (Remarks to the Author):

I think the authors did a great job further improving their manuscript. At this stage I only have a few very small comments left (see below), which do not stand in the way of recommending this manuscript for publication in Nature Communications. I leave it to the authors to decide whether or not to take them into account.

Comments:

- Line 215. I do not understand what is meant by "passing the filtering by varying management". Other than that, it is interesting to see that the positive relationship between plant species richness and plant P stocks in the diversity experiment becomes negative in the agricultural grasslands, and that this is not caused by the agricultural grasslands having lower diversity of a few highly competitive species, as I suggested. However, the explanation involving plant biomass is still not entirely clear to me. I can follow that plant biomass is related to plant P stocks, and that because management intensity increased plant biomass, it also increased plant P stocks. That would be the blue direct arrow in Fig. 1b. I can also understand that high management intensity lowers species richness, but then why is lower species richness resulting in higher P stocks? Your explanation in the rebuttal letter suggests that fertilization effects overrule diversity effects, but would such fertilization effects not already be captured by the direct arrow in Fig. 1b between management intensity and plant P stocks? The model explicitly teases out the relationship mediated by plant species richness. My point is that effects of missing variables (such as fertilization) should be captured by the direct path, unless the missing variable is highly correlated with plant species richness, and similarly responds negatively to management intensity and itself negatively relates to plant P stocks. In short, you convinced me that my hypothesized explanation is not valid, but you have not convinced me yet of any potential mechanism behind the pattern you observe here.

- Line 478. I appreciate your explanation as to why you decided to use averages across both years. You could actually use the information you provide in the rebuttal letter that variables were significantly correlated among both years to argue why you used averages. To me, the fact that they are correlated is an indication that year-to-year variation is not huge and therefore data can be collapsed into a single average over time. I leave this decision to the authors.

- Table S2 and S3. I suggested earlier to change "biomass" to "plant biomass" for clarity, and the authors say they did so, but it still seems to show as just "biomass" in my copy of the revised manuscript.

Response to reviewers.

Here we report our responses to the reviewer concerns in black following their comments in grey italics.

REVIEWER COMMENTS

Reviewer #1 (Remarks to the Author):

Nature Review

The paper written by G. Duveiller et al. on "Widespread cloud enhancement adds further value to the world's forest" present an ambitious scientific work to evaluate at a global scale the possible impact of afforestation on low cloud cover. The conclusions are that afforestation would lead to an increase of low clouds cover that would in turn increase the Earth surface albedo above the forests and have a cooling effect. It also indicates that needleleaf trees have an amplified effect on these cloud covers because of biophysical effects on clouds formation. The work is well descripted and most of the hypotheses and methodological choices are well justified using the most recent literature in many different fields (atmospheric science, land cover change monitoring, biology…) giving to the work a real interdisciplinary framework. The work is of particular interest to evaluate possible solutions to the on-going climate change process giving a particular look at land-based GHG mitigation options. The datasets are well documented as well as the overall methodology. The decision to verify the nature of the trends of low cloud cover effects after afforestation using different datasets (satellite-based and ground-based) and different resolutions is also relevant since the authors decided to prioritize an observation-driven assessment that can serve to other scientific groups as a benchmark and framework for climate modelling.

In general, the quality of the paper is really high and I would agree with most of its conclusions considering that the methodology is particularly convincing. This said I would suggest that some of the explanations given to the observations are better explained.

We thank the reviewer for the praise and have tried to add more detail were possible to improve the text as suggested.

The introduction would benefit greatly from a short explanation on the life cycle of the clouds (at different time scale, daily, monthly…). Cloud formation should be explained as well as other steps of cloud life cycle.

We have partly reorganized the introduction to accommodate more explanation of the formation of clouds, and more particularly on the diurnal life cycle of low level clouds based on the paper by Eastman & Warren 2014. Convective cloud formation was already

partly described when describing the interplay between trees and clouds, but we have also amended that part based on the recommendations of reviewer 2.

Also effect of presence of low-level clouds on the cloud formation of deeper convection should be mentioned particularly for ITCZ regions.

Following this request/suggestion from the reviewer, we have now added a mention about how low-level clouds are thought to affect tropical convection, citing the paper of Fermepin & Bony (2014). We have kept it in the introduction when presenting the radiative effects of clouds. We must say that we have generally abstained from speculating how the low-level clouds that are linked to forests might affect other types of clouds. This is mostly because our tools (i.e. satellite observation driven analysis) is ill-equipped to really analyse such effects. For instance, regarding our space-for-time methodology, our general position is that other clouds (both low level stratus clouds or mid to high level clouds) would not be strongly spatially correlated to the underlying landscape features, and would thus not be “visible” by the method. They would generate white noise. We have added more details on this in the methods part to be clearer. This does not prevent, as the reviewer suggests, that low level cumulus clouds might aggregated and affect deep convections, and thereby affect differently the full energy balance. But as mentioned already in our conclusion, we consider that further investigation of the full radiative effect would require the use of deterministic physically-based models.

The assumption that in some cases deforested areas can lead to a stronger cloud cover without taking in account irrigation is problematic since it changes the overall scenarios. This issue should be reinforced by other reference or complementary regional data if existing.

There might be a misunderstanding here. We do not assume that irrigation is not taken into account. On the contrary, the effect of irrigation is part of the picture, as it is “seen” by the satellite measurements. The results we present depend on the actual cropping practice in vigour at the regions that are observed. Basically, if we consider potential deforestation in areas were irrigated crops are presently common, the estimation of the effect of deforestation on cloud cover will be that of a transition from forest to irrigated cropland. If we consider an area without irrigation, the transition has to be assumed as going from forest to non-irrigated cropland. The same is true for the inverse transition of afforestation. We hope our rewording in the text has helped clarifying this point. As a reminder, we stress again that none of these transitions need actually to occur for our diagnostic to work, as we always do a space-for-time substitution.

Also, there is no information on wetlands and coastlands where water availability is high and with possible strong border effects as we can see on the map of Sri Lanka.

Indeed, we had not gone into these details to avoid overloading a manuscript that seemed already very dense, but we have now amended this. Wetlands are actually considered as

a separate class in the land cover characterisation we use, along with other classes we did not mention such as shrublands and savannas. These complement the 3 classes we did talk about in the manuscript (deciduous forests, evergreen forests, and herbaceous vegetation) in defining the possible end-members of the space-for-time unmixing procedure, i.e. the potential source and target classes in the possible vegetation transitions that we can consider. This means that the result of the procedure yields changes in CFrC values for all transitions among these 6 classes. To simplify the message in the paper we explicitly decided to focus only on the transitions involving Forests and Herbaceous land cover. However, this means that effects of Wetlands are effectively factored out from the results we show (as far as the wetlands are correctly mapped in the original land cover map).

Coastlines are also factored out from our results but by another mechanism. The parametrization of our space-for-time unmixing requires that 60% of the 7 by 7 pixel window be covered by valid pixels. Water is a criterion of exclusion. As a result, when the moving window approaches the coast, once there are more than 20 water pixels, it is automatically eliminated. In practice this means that there is a buffer around coastlines for which the values are ignored, precisely to avoid these areas where the cloud formation may be dominated by the sea. With possibly 20 pixels in the window coming from the sea, it is true that some residual coastal effect could still occur, but the value would actually be calculated from the rest of the pixels which are mostly several kilometres away from the coast, so we consider that overall the current parametrisation should still mitigate a large part of coastal effects across the globe. We have added these details in the text.

Regarding the details in Sri Lanka, indeed, this might be a place where the coastline filtering might be sub-optimal. We have mentioned that further refinements could be made in future work to better control the coastal effect. As a general remark, we have abstained ourselves from overly interpreting small regional details and have focused on the larger, global or continental patterns, as we deem the methodology should be better adapted to the regional context to be more precise (i.e. with adapted parametrization, with finer land cover maps and cloud data for instance).

There is also something appearing as a border effect on the western part of the Xingu protected indigenous territory in Mato Grosso (Brazil) that would be interesting to comment since some researchers consider this region as essential for the climatology of Mato Grosso in terms of clouds formation.

The prominence of the relatively intact Xingu territory within the Mato Grosso state can does probably affect the region’s climate in terms of cloud coverage, but we are unsure whether we can say much more with our current analysis. As with the case mentioned above with Sri Lanka, we prefer not to speculate too much on the local/regional effects with our current implementation, which was never meant to look at patterns at such a level of detail.

But in general I would suggest a rapid publication of this paper that will certainly enhance the scientific dialogue on these urgent issues.

Minor recommendations :
P.20 – line 339 : It would be similarly hard to quantify the added value of potential afforestation in terms of precipitation without using to some modelling framework.
- delete "to"

Done

P31 – L507 - "thus implicitly assuming there is only limited wind during those few hours." Do you have any scientific reference to justify better your assumption in the methodology on the clouds lateral movements? Please provide some numbers to facilitate the calculous (resolution/spatial window, time difference between image acquisitions, convective cloud normal speed, existing wind speed data…). This is before looking at the sensitivity that answer only in part to this assumption.

We realize that our formulation here could be improved. We previously stated that "these are mostly convective clouds generated during the morning", but to be more precise, it is the conditions of increasing heat load occurring in the late morning that facilitate the generation of clouds, which actually form later. As reported by ground level climatologies, the peak of presence of low level cumulus clouds generally occurs a little after noon (Eastman and Warren, 2014), precisely at the time of the satellite overpass. Furthermore, this seems to be very consistent across latitudes and seasons (Eastman and Warren, 2014). The idea that clouds would form in the morning and have the opportunity to move around for 3-4 hours before we detect them at noon is therefore incorrect, and we have rectified the text to avoid such confusion.

In any case, it is also important to consider is that convective cloud formation occurs typically in situations of high pressure when there is limited wind. At the boundary layer, around 850hPa, we would expect wind speeds of 2-3 ms^-1, which does not leave much ground for movement across pixels of several km large when we consider we are looking just about the same time (circa 13:30) as when they see their peak formation.

It can further be stressed that the fact that a consistent signal does show up in many parts of the world is already a sign by itself that the effect is real despite possible lateral wind movements. That is because if the clouds have moved sufficiently to be far beyond the original land cover over which it was generated (and be instead over a random land cover), then the result of our unmixing methodology would be white noise. Therefore, if anything, the presence of small lateral wind movements would have the effect of diluting our signal, so our signal could actually be an underestimation.

To better reflect all these points, we have re-organised our section on our assumptions and even placed it before the data description, as we find it flows better this way.

Reviewer #2 (Remarks to the Author):

Jeffrey M. Freedman: Reviewer comments on "Widespread cloud enhancement adds further value to the world's forests

This paper set forth the hypothesis and presents an analysis that afforestation enhances the formation and presence, on a global scale, of low-level (boundary layer) clouds. For the most part, the analysis is presented in a sound, consistent manner, and at a minimum demonstrates the influence of forests on surface-atmosphere exchange processes and the presence of boundary layer cumulus clouds. In general, the authors found that the presence of forested land (or afforestation) increases the frequency of low-level cloudiness, consistent with the findings of other studies. Overall, the paper is well-written, methodologies are carefully laid out (mostly in the supplemental material), graphics are clear and easy to understand, and the discussion and conclusions are well posed. The paper presents work that will be of value to current and future research efforts investigating forest-atmosphere exchange processes on regional to continental scales.

General comments:

For the link between boundary layer cumulus clouds (BLcu) and surface atmosphere interactions, see Freedman et al. (2001) for seasonality of BLcu and Freedman and Fitzjarrald (2001) for modification of local and regional heat and moisture budgets by the forest through BLcu (seasonal equilibrium).

We thank the reviewer for providing these valuable references that we had overseen in the first draft. We have now included both references in the introduction when discussing the mechanisms of how low-level convective clouds are formed above the forest.

One limitation is that the study relies heavily upon satellite observations, particularly once-daily observations (around 1330 - 1400 LT) of satellite-derived (from MODIS [Aqua]) cloud fraction with a resolution of approximately 2 - 5 km (0.02o — 0.05o). A comparison with the surface stations used in the study (that is, what is the diurnal variation of low clouds at representative sites would indicate any temporal bias in the satellite data vis-a-vis cloud fraction reports.

The comparison we did between the Aqua satellite estimations and those from the ground stations (shown on Fig 3c) is done for the specific time of 14:00, a time in the day that seems to show peak presence of cumulus clouds pretty much across the whole land and for all seasons (see Eastman & Warren 2014, which we now included in the paper). Fig 3b shows that the patterns (at least here for Europe) seem quite consistent at different times (e.g from 10:00 to 16:00 at least). This would suggest that there is no such temporal bias, at least for Europe. Of course, there is still a (non-temporal) bias in absolute terms as the

values (in Fig 3c) do not match, but this is most probably due to the different approaches used to get the corresponding estimates (i.e. from satellite near nadir top-down observations and from ground hemispheric bottom-up observations).

And note that BLcu typically scale to less than 1 km (even below 0.5 km), which can bias satellite reporting of cloud fraction.

We agree, cumulus clouds would generally be smaller that 1 km across. However, while the ESA CCI cloud product we use is at either 0.05dd or 0.02dd of spatial resolution (i.e. ~5km or ~2km), it does not mean that it is not sensitive to sub-grid cell variations. In fact, the product is not binary (i.e. cloud or not cloud) but provides a continuous values based on sub-grid cell objects which are detected at finer spatial resolution. The original MODIS data ingested in the ESA CCI cloud product are 1km in spatial resolution, but actually 2 of the 5 bands they use are detected by the satellite using even finer bands which have a spatial resolution of 250m before being averaged to 1km for ease of use. Furthermore, clouds (and cumuli clouds in particular) are so bright with respect to most backgrounds that their mere presence within a large pixel is sufficient to cause a detection. We are therefore confident that the product is sensitive to a large proportion of the clouds of concerns and that such a bias would remain relatively small. We have changed the text in the Material and Methods section, particularly where we describe the datasets, to reflect these points.

Note that low-level (BLcu) clouds are rooted to the terrain. Although the authors have filtered data to remove complex terrain from the analysis, subtle differences in terrain elevation and land-use can result in differences in cloud climatology.

Yes, we agree, and this is why we have spent quite some effort in removing topographical effects. Please note that there is a triple criteria to remove topographical effects, and that it goes beyond simply removing very complex terrain. We had previously thought of keeping this description to a minimum (referring instead to the previous study), but how now decided to include more details about this topographical filtering for completion. We similarly included more detailed information on how we calculated the pair-wise occurrence, another important filtering step ensuring the quality of the results.

But we agree that we cannot guarantee that all subtle differences can be accounted for. This is partly why we also re-did the analysis using a second method in which we only looked at areas were land cover change has occurred (see Figure 3, formerly Figure 2). While that method is limited by the fact that there is only limited land cover change across the world, the fact that we also see the same patterns gives us confidence that despite potential residual terrain effects, our space-for-time method is providing a reliable diagnostic of what potential afforestion entails.

Given the limited period of record (apparently from the supplemental document 7 - 10 years?), is that a long enough POR to determine whether forest regrowth (afforestation), and not inter-annual variability (or a combination thereof) is contributing to apparent trends in cloud fraction?

The period of record used in the paper was limited by the availability of the specific ESA CCI cloud MODIS Aqua dataset, which is available only for the period 2004-2014, thus consisting of 11 years. We do believe this period should be sufficient for our purposes. Since we are not sure whether the reviewer is rising this remark regarding the main 'space-for-time substitution' method employed in this paper, or for the alternative method based on real changes (which is only used as an extra source of confirmation that the signal is correctly attributed as it has already stated limitations), we here discuss the implication of the length of the archive for both methods.

For the main methodological approach, we make a space-for-time substitution to estimate the effect of afforestation, meaning that we do not wait for trees to be planted to see their effect on clouds. The concern of the reviewer is therefore not really applicable as we do not NEED afforestation to occur. Furthermore, we are never looking at temporal trends, but only seasonal cycles and spatial gradients. Regarding interannual variability, we work at monthly frequency, which already filters out the day-to-day variability. We then proceed to doing a temporal average to result in a single value for every month at every gridcell. This 'climatology' should also remove the effect of interannual variability to a considerable extent (although it would indeed be preferable to have more than 11 years).

For the alternative method based on actual change, the period of 11 years may indeed be problematic for detecting afforestation/forest regrowth, as some forests may take longer than this to be fully established. However, the method is symmetric with regards of the transition, meaning that the effect of afforestation on cloud cover is inverse in sign with the effect of deforestation. The main statistic is indeed extracted from observations of forest loss, which are more clearly detectable even within the 11-year period. With regards to the limited number of years to statistically derive the information, this method actually uses all possible combinations between any two years within the 2004-2014 period, and this is done separately for each month. This inflates the sample number considerably, allowing for a more robust retrieval of the estimation of the cloud effect. We realize some of details were missing in our original manuscript and have now amended it accordingly.

It may make sense to move some of the supplemental material to the main narrative, such as identification of the data sets used, periods of record, and basic methodologies regarding cloud retrieval.

This is partly due to the format of the journal, that requests Material and Methods placed at the end. But we do agree some more technical information might be warranted in the main narrative, and we have thus tried to do so to a point.

Specific comments:
Page 5, lines 86 - 88: regarding the statement "Forests have larger net radiation and surface roughness than open lands, favouring a better exchange of heat, moisture and momentum with the atmosphere, which in turn generates a stronger convection and a deeper atmospheric boundary layer." Since forests contribute substantially to the local and regional moisture budget, resulting in lower Bowen ratios, more energetic thermal may tend to be in areas with locally higher sensible heat flux—the key is the balance between the temperature and moisture tendencies and where the local/regional lifting condensation level is (see Freedman and Fitzjarrald 2001).

We have clarified this point and added the reference appropriately

Page 6, lines 123 - 125: the statement "However, it is important to note that by design the methodology to extract these local changes in CFrC are only sensitive to low level clouds (typically cumulus clouds), and thus all changes in CFrC shown should be considered as changes in low level clouds only." It is unclear that one can make this statement conclusively (although the top of page 13 discussion does allude to indirect evidence of an increase in the sensible heat flux—but this is questionable given afforestation would seem to favor a lowering of the Bowen ratio given the increase in LE resulting from increased evapotranspiration during the growing season).

The statement stems more from a logical deduction of what the method can or cannot 'see'. We assume that the vegetation predominantly affects cloud fraction cover by generating boundary layer convection. While these cumuli could self-aggregate and affect deep convection at a later stage (as suggested by Reviewer 1), we consider that it is highly unlikely that the resulting patterns would be strongly related to the fine scale structure of the land. Rather, they would appear random with respect to the land within our local 7 by 7 pixel windows, and the method would therefore not 'see' any signal. The fact that there is a signal indicates that there is a spatial correlation within our local windows between cloud fraction and land cover. We have added more clarifications in the text and palced more emphasis to this in our "assumptions" section.

Page 12, lines 184 - 187: The statement "While in some places cloud occurrence could be limited by the heating needed for uplift, in some it could be limited by moisture supply. The relative importance of various different factors would need dedicated studies based on modelling experiments…where different factors can be isolated." Much of this was discussed and analyzed in Freedman and Fitzjarrald (2001). Forests tend to foster an equilibrium between the surface and the atmosphere, reaching an RH equilibrium (and steady-state LCL) during the growing season.

Again, thank you for this reference, which we have now added, and our apologies for not having spotted it before. Our comment regarding the fact that modelling experiments

would be needed was meant considering all geographic locations together across the globe, which would be difficult based on ground data as in the study Freedman and Fitzjarrald (2001). We have added “across different geographical locations” in the phrase to make that clear.

Page 13, lines 206 - 208: Beginning “However, in situations in which there is no change….” See above comment.

We agree with the comment above: afforestation will typically lower the Bowen ratio. The comment we wrote here refers specifically to the observation within Figure 5c (formerly Figure 4c) that when there is no change in the combined ground + sensible heat flux (along the vertical axis), comparing two types of regions, one in which higher increases in latent heat flux are observed and one in which lower increases in latent heat are observed, the former shows a higher increase in CFrC. This at least seems to be the case from the range of Delta_HG from -5 to +10 Wm-2. But rather than going in the specifics of the figure, we have reformulated this part to rather state that this analysis (shown in Figure 5, formerly Figure 4) basically confirms that both processes (injection of moisture by LE, and bulk air displacement by H) are correlated with higher cloud fraction cover.

Page 15, lines 229 - 230: “…we do not isolate this signal in our dataset as we make no distinction between irrigated and on-irrigated crops.” Note that irrigation plays a very large role in transpiration and evapotranspiration in the US Midwest and Great Plains (e.g., Alter et al. 2015).

We realize that the formulation here might make things a bit confusing. We agree that irrigation has a very large role. What we meant is that the method does not isolate irrigation effects explicitly in our analysis, but the effect of irrigated crops is still present in the signal that is extracted by the space-for-time substitution. One just needs to know that when one looks over areas where irrigation is dominant, the signal relates to irrigation. We have tried to clarify by removing this mention of “the signal” here, and have clarified that irrigation is present in the signal in several other parts of the manuscript .

Page 17, lines 288 - 291: The statement “According to the first assessment, afforestation and forest management in Europe during the past 250 years have not mitigated climate warming…partly because these operations involved a transition from brighter broadleaf species to darker conifer forests.” Is it possible (although the cited article does not discuss this) that much of the warming may be due to the more rapid rise in night time minimum temperatures (more low level clouds; e.g., Dai et al. 1999).

This is an interesting point and worth clarifying. However, we find that it may be outside of the scope of the specific discussion we are doing in that paragraph.

References

Alter, R. E., Y. Fan, B. R. Lintner, and C. P. Weaver, 2015: Observational Evidence that Great Plains Irrigation Has Enhanced Summer Precipitation Intensity and Totals in the Midwestern United States. J. Hydrometeor., 16, 1717–1735, https://doi.org/10.1175/JHM-D-14-0115.1.

Dai, A., K. E. Trenberth, and T. R. Karl, 1999: Effects of Clouds, Soil Moisture, Precipitation, and Water Vapor on Diurnal Temperature Range. J. Climate, 12, 2451–2473, https://doi.org/10.1175/1520-0442(1999)012<2451:EOCSMP>2.0.CO;2.

Freedman, J. M., and D. R. Fitzjarrald, 2001: Postfrontal Airmass Modification. J. Hydromet. 2, 419- 437.

Freedman, J.M., Fitzjarrald, D.R., Moore, K.E. and Sakai, R.K. 2001: Boundary layer clouds and vegetation–atmosphere feedbacks. J. Climate, 14, 180–97.

Reviewer comments, second round –

Reviewer #1 (Remarks to the Author):

Dear Authors,
Thanks for considering the review comments and improving your paper according to the different edition requests. Your paper was greatly improved and I agree for its immediate publication. Congratulations for this achievements, very cordially

Laurent Durieux

Reviewer #3 (Remarks to the Author):

Review for Nature Communications Article NCOMMS-20-37271A –
Widespread cloud enhancement adds further value to the world's forests

The study analyzes, using various types of in-situ and satellite datasets, the impact of forests on shallow cumulus clouds as compared to clear land. Authors use ~10 year records of various datasets to correlate cloud fractional cover with either forest or grassland land cover. The analysis is performed over the global domain and also over continental and regional scales utilizing various datasets. The analysis shows a positive correlation of shallow clouds with forest cover in most parts of the globe analyzed and during most seasons except summers in some regions. Authors have also analyzed components of the surface energy balance to explain the land cover-cloud coupling in general on the global scale. The main focus of the study, however, remains the understanding of how afforestation can impact local shallow cloud cover. Results support the notion that forests could possibly provide a buffer against climate change by not only acting as carbon sinks but also by reducing planetary albedo through their impact on low level cloudiness.

Overall I find the analysis of land cover-cloud coupling to be sound and careful. The results are also quite interesting, intriguing and important and will interest a broader scientific community. However, the analysis of underlying processes, although not the focus of this study, has not been carefully performed and presented. Because this analysis is not central to the study, and because there are no apparent conclusions reached regarding underlying processes, it is hard to justify why this section is included in the manuscript. In my point of view this section hampers the quality of the rest of the manuscript. I would suggest either a complete removal of this section or a major reassessment of the arguments presented there. The manuscript could be recommended for publication once this issue is addressed. I have provided my comments below.

Note – As per my understanding, authors have addressed most comments by reviewer #2 from the previous round of review, except specific comments 1 and 2.

General Science Comments –
1. Although most of the regions analyzed show a contiguous signal, some regions have a mixed signal with very large differences of opposite sign next to each other (most regions of Africa, most pixels over south America in Figure 1 a-d). This random signal I find quite disconcerting. The average of these pixels will indeed have some sign, which can then be analyzed, but then how believable is that signal? It will be helpful for the reader if the authors comment on this in the manuscript.
2. I find it better to represent differences as percentages of the mean to provide the sign and magnitude of change together with perspective as compared with the average conditions. Percentage differences in most figures will help include more information in the same space. Authors can consider making this improvement.
3. Line 156 – 5% differences seem to be quite small. It will be helpful if the maps and other figures show percentage differences rather than absolute differences.

4. In the land cover classification scheme, authors have put irrigated cropland under herbaceous vegetation. This can apparently impact the sign of the cloud cover change over open land and nearby forests. Although there is no inherent issue with this study design, but clubbing these types of land cover will impact the analysis of underlying physical processes.
5. Discussion around Figure 5 – This analysis, in general, is not very rigorously performed which substantially brings down the quality of the manuscript. First of all, such an analysis can not be performed for all grid points spanning so many different climatic zones and myriad vegetation types. For example, apart from surface heat fluxes, land-atmosphere coupling could come into play due to other reasons as well which will be completely missed by this analysis. Further, such analysis would be incomplete without any information about the time of the analysis. No conclusions can be drawn from this analysis if the components of the surface energy balance do not represent afternoon conditions. Authors should clarify that. In my opinion a common underlying process cannot be and should not be aspired for. Hence this analysis does not add constructively to the rest of the manuscript.
6. line 219 – Usually forests are associated with higher LE and higher shallow cloud cover. This is usually not because forests cause increased convective triggering but because there is enough transpiration to provide moisture for cloud formation. Moreover high humidity also provides conducive conditions for convective triggering. So it is really interesting that high increase in CFrC is observed with low increases in LE.
7. Lines 219-227 – The discussion in these lines is a bit confusing. It is not clear whether a high increase or a low increase in LE is correlated with high increase in CFrC.
8. Lines 232-236 – I find this discussion quite vague. why is the bl dry even though the LE is the same? why does a dry bl favor greater momentum exchange? and why is this important?
9. Lines 236 – 244 – So is the high CFrC over open land due to radiation fog? In that case should the data from these regions in the said season be included in the analysis? There is potential for confusing processes behind increasing cloud cover and those behind increasing fog.
10. Lines 252 – 254 – This seems very unlikely because during the monsoons local vegetation-atmosphere coupling is expected to be very weak.
11. Lines 318-322 – This comes across as an overstatement because no analysis focused on evergreen conifers and their roughness length, albedo, stomatal conductance has been presented in the manuscript. This is only the author’s conjecture.

Specific Comments –
1. It will be beneficial to have some important details of the methodology in the main text. These details will include the datasets used, time period considered, temporal averages performed, spatial averaging.
2. Lines 109-113 – Earth system models do not fully represent all land-atmosphere interactions. Also these endeavors are not just limited by the computational difficulty to finely represent these complex biophysical effects at kilometer scales.
3. Line 225 – layer and to the increased bulk … of the forests. This sentence is not clear.
4. Lines 309 – 312 – Ths sentence is unclear.
5. Figure 1, last line – change ‘were no’ to ‘where no’.
6. Line 258 – change ‘that C3’ to ‘than C3’.
7. Line 264 – change ‘an heterogeneous’ to ‘a heterogeneous’.
8. Line 284 – change ‘value of forest’ to ‘value of forests’.
9. Line 400 – change ‘assumption it that’ to ‘assumption is that’.

Response to reviewers.

Here we report our responses to the reviewer concerns in black following their comments in grey italics.

REVIEWER COMMENTS

Reviewer #1 (Remarks to the Author):

Dear Authors,
Thanks for considering the review comments and improving your paper according to the different edition requests. Your paper was greatly improved and I agree for its immediate publication. Congratulations for this achievements, very cordially

Laurent Durieux

Thank you, and thank you for your help in improving the manuscript.

Reviewer #3 (Remarks to the Author):

Review for Nature Communications Article NCOMMS-20-37271A –
Widespread cloud enhancement adds further value to the world's forests

The study analyzes, using various types of in-situ and satellite datasets, the impact of forests on shallow cumulus clouds as compared to clear land. Authors use ~10 year records of various datasets to correlate cloud fractional cover with either forest or grassland land cover. The analysis is performed over the global domain and also over continental and regional scales utilizing various datasets. The analysis shows a positive correlation of shallow clouds with forest cover in most parts of the globe analyzed and during most seasons except summers in some regions. Authors have also analyzed components of the surface energy balance to explain the land cover-cloud coupling in general on the global scale. The main focus of the study, however, remains the understanding of how afforestation can impact local shallow cloud cover. Results support the notion that forests could possibly provide a buffer against climate change by not only acting as carbon sinks but also by reducing planetary albedo through their impact on low level cloudiness.

Overall I find the analysis of land cover-cloud coupling to be sound and careful. The results are also quite interesting, intriguing and important and will interest a broader scientific community. However, the analysis of underlying processes, although not the focus of this study, has not been carefully performed and presented. Because this analysis is not central to the study, and because there are no apparent conclusions reached regarding underlying processes, it is hard to justify why this section is included in the manuscript. In my point of view this section hampers the quality of the rest of the manuscript. I would suggest either a complete removal of this section or a major reassessment of the arguments presented there. The manuscript could be recommended for publication once this issue is addressed. I have provided my comments below.

We thank you for your review and the constructive suggestions. We agree that the part describing the underlying processes is not the main strength of our study. However, removing the whole section entirely seems to us excessive as it would leave no interpretation or explanation whatsoever of the patterns we expose. We understand that the main criticism is on the analysis of the drivers summarized on our (former) Fig 5. We have thus removed that specific part from the main text, and we have tried to adapt the tone of the rest of the section to be more descriptive than affirmative. The idea is to present to the readers some of the mechanisms that might explain the patterns behind our findings, rather than to quantify the underlying mechanisms (which we agree it is beyond the scope of this work). We believe that, as a result of this suggestion, we have considerably improved the flow of the manuscript.

Note – As per my understanding, authors have addressed most comments by reviewer #2 from the previous round of review, except specific comments 1 and 2.

We are unsure about why Reviewer 3 considers we have not addressed these two specific comments.

In the first comment we explicitly wrote a phrase paraphrasing what Reviewer 2 was pointing out:
“Furthermore, research suggests that, thanks to a feedback between cloud generation and its subsequent effect on the in-coming radiation, forests tend to foster the establishment of an equilibrium between the temperature and moisture tendencies ensuring that these cloud-forming conditions endure ” and we cited the appropriate reference (Freedman and Fitzjarrald 2001).

Regarding the second specific comment 2, which questioned how confident we were on our statement that by design, our method should be sensitive mostly to low-clouds, we had already added a detailed justification in our assumptions in the Methods section.

General Science Comments –
1. Although most of the regions analyzed show a contiguous signal, some regions have a mixed signal with very large differences of opposite sign next to each other (most regions of Africa, most pixels over south America in Figure 1 a-d). This random signal I find quite disconcerting. The average of these pixels will indeed have some sign, which can then be analyzed, but then how believable is that signal? It will be helpful for the reader if the authors comment on this in the manuscript.

We agree that there are parts of the world that might appear to have a more erratic behaviour at some specific times. However, we disagree that this concerns “most” of Africa and south America as the reviewer implies. Our figures show some clear macro-patterns over these continents, despite some local variability. We do not expect to have a clear and fully spatially consistent signal everywhere, as the process of cloud formation linked to the land surface will be inherently difficult to catch everywhere with the same efficiency using a single method with its set of assumptions. We have tried to make that clearer by adding a sentence in this regard in the beginning of the results, which itself refers to the descriptions of the assumptions made in the Methods section.

2. I find it better to represent differences as percentages of the mean to provide the sign and magnitude of change together with perspective as compared with the average conditions. Percentage differences in most figures will help include more information in the same space. Authors can consider making this improvement.

We agree that percentages provide a useful metric as it is more easy to relate to. This is why we had included such an equivalent of figure 1 with percentages in the supplementary material. Following the reviewer’s suggestion in the revised version we have thus switched and placed the figure with percentages as the main figure in the main text, and relegated the “absolute” figure to the supplementary material’

However, it is not so trivial to calculate this value in terms of percentage for all the analysis/images. In fact, it is not obvious if this should be done in relation to the average cloud cover over the forests or the average cover over the herbaceous land cover. Getting that value prior to our space-for-time approach is not easy either, as we are dealing with mostly mixed pixels and therefore the estimation of these averages would need a complex and uncertain unmixing of the average signal. What we did in our Figure 1 was to work at the 1dd cell scale and divide our estimates of absolute change that are aggregated to that level by the average cloud cover over that cell. This reference cloud cover is therefore not exclusive of the forested or non-forested area, and furthermore, it would be very depending on how much forest there

is in the first place. Therefore, we opted to change to percentages only for Fig.1. but kept the rest in absolute terms.

3. Line 156 – 5% differences seem to be quite small. It will be helpful if the maps and other figures show percentage differences rather than absolute differences.

We have changed the numbers to percentages where possible.

Do note that even if 5% might seem small, such an increase in cloud cover could substantially affect the planetary energy balance. The ratio of radiation arriving at the surface between a sunny day and a cloudy day can be, roughly speaking, 5 to 1. That 5% increase could thus have a much stronger effect. We do not venture to quantify here the consequences of such an effect as it is beyond our scope and would probably not be currently doable based on observational data-streams only. Such a calculation would require a process-based modelling exercise where there is a full control of all variables involved.

4. In the land cover classification scheme, authors have put irrigated cropland under herbaceous vegetation. This can apparently impact the sign of the cloud cover change over open land and nearby forests. Although there is no inherent issue with this study design, but clubbing these types of land cover will impact the analysis of underlying physical processes.

We agree, irrigation is potentially a clear driver for cloud formation. We would have liked to include such a class explicitly and individually in our analysis. However, to do so, we need to be sure that this specific class is clearly identified accurately across the globe. Basically, we do not trust that the land cover product we use does that correctly and consistently everywhere for this specific class. Several regions around the world that do have irrigation are not labelled as such in these maps. Also, our method is penalized by having many classes of sparse coverage, making it more important to “club” classes together when possible. The compromise was to have this more general “herbaceous class” knowing that in areas dominated by irrigation, the signal can be interpreted as involving the class “irrigated” (as already mentioned later in the “Considerations on the space-for-time method” sub-section of the Methods). In any case, we added an extra line stating why we do not use the irrigation class individually.

5. Discussion around Figure 5 – This analysis, in general, is not very rigorously performed which substantially brings down the quality of the manuscript. First of all, such an analysis can not be performed for all grid points spanning so many different

climatic zones and myriad vegetation types. For example, apart from surface heat fluxes, land-atmosphere coupling could come into play due to other reasons as well which will be completely missed by this analysis. Further, such analysis would be incomplete without any information about the time of the analysis. No conclusions can be drawn from this analysis if the components of the surface energy balance do not represent afternoon conditions. Authors should clarify that. In my opinion a common underlying process cannot be and should not be aspired for. Hence this analysis does not add constructively to the rest of the manuscript.

We agree that this analysis is perhaps the weakest part of the manuscript. We had kept it because during previous iterations of the review process (in a previous journal) we were asked precisely to strengthen the explanation of the drivers. We do agree, however, that as is it does not add constructively to the main flow of the manuscript. We have therefore removed it from the main text, as suggested. We believe the result is a streamlined main text that flows much better.

Having said that, we consider that despite its shortcomings, this part can still be informative, and that sooner or later someone would be intrigued in putting these datasets together and try to draw conclusions from it. Therefore, we have decided to retain the figure and the brief analysis as a complementarity analysis at the end of the method section, but clearly stated the shortcomings.

6. line 219 – Usually forests are associated with higher LE and higher shallow cloud cover. This is usually not because forests cause increased convective triggering but because there is enough transpiration to provide moisture for cloud formation. Moreover high humidity also provides conducive conditions for convective triggering. So it is really interesting that high increase in CFrC is observed with low increases in LE.

While higher transpiration above forest definitely provides more moisture that is susceptible to condense, there is also another process that can come into play: the height of the boundary layer. This in turn can be higher for a same amount of LE if there is more sensible heat, perhaps because of a darker canopy that increases net radiation, for instance.

We agree that it is interesting that we observe this, which is partly why we think it is worth to keep this figure as a complementary information after the caveats and shortcomings are clearly stated, as we now do in the revised text.

7. Lines 219-227 – The discussion in these lines is a bit confusing. It is not clear whether a high increase or a low increase in LE is correlated with high increase in CFrC.

We have reworded and simplified this part, hopefully reducing the confusion.

8. Lines 232-236 – I find this discussion quite vague. why is the bl dry even though the LE is the same? why does a dry bl favor greater momentum exchange? and why is this important?

We probably should have formulated differently to increase clarity. Even under equivalent LE, the boundary layer can be drier if the height of this layer in larger. Such a deeper boundary layer would dilute the moisture content in a greater volume, and thus could be drier. And the boundary layer can be deeper is there is more sensible heat (for an equivalent amount of LE). We believe this might be occurring over dark needleleaf forests in winter conditions (when they do not transpire much), when comparing them to adjacent snow-covered fields and grasslands.

9. Lines 236 – 244 – So is the high CFrC over open land due to radiation fog? In that case should the data from these regions in the said season be included in the analysis? There is potential for confusing processes behind increasing cloud cover and those behind increasing fog.

Indeed, as we write in our text, we think radiation fog is a plausible explanation for the patterns we see in this part of the world and for this season. We had considered trying to separate out the parts that we suspect are due to radiation fog, but we did not for 2 reasons: (1) we think it is better to remain transparent and show that we do see this effect instead of masking it out; and (2) we do not have a straightforward way to entirely separate this effect just from the data.

10. Lines 252 – 254 – This seems very unlikely because during the monsoons local vegetation-atmosphere coupling is expected to be very weak.

We agree that local vegetation-atmosphere coupling during the monsoon should be weak. In fact, we have revised this part of the text to actually highlight how this period of reduced coupling clearly explains the seasonal pattern we see for the changes in CFrC over the Indian subcontinent, which is characterized by a clear dip in magnitude during the monsoon period only to rise back again as the rainy season recedes.

However, there is still an intriguing fact that during June the sign of the CFrC change actually reverses. We speculate that this might still be resulting from the fact that without water stress, herbaceous vegetation might transpire more as they have a

lower Bowen ratio (given their lower hydraulic and stomatal resistance), but we also stressed research is needed to clarify this.

11. Lines 318-322 – This comes across as an overstatement because no analysis focused on evergreen conifers and their roughness length, albedo, stomatal conductance has been presented in the manuscript. This is only the author's conjecture.

We have not explicitly used these variables quantitatively in the study, mainly because (aside from albedo) these are currently not available from observational data streams (such as satellite imagery). However, the separation into two forest classes from the land cover perspective is robust, reason why we explicitly used this class separation in our un-mixing approach (unlike with the irrigation class which we decided not to trust, as mentioned previously). And there is a general consensus that needleaf forest do typically have lower albedo, lower stomatal conductance and larger roughness lengths. Therefore, we do not think that bringing these as possible explanations is an overstatement. To ensure our message remains prudent we have revised the wording here, saying this "could stimulate cloud formation".

Specific Comments –
1. It will be beneficial to have some important details of the methodology in the main text. These details will include the datasets used, time period considered, temporal averages performed, spatial averaging.

In general, we have tried to stick to the journal's format asking for these details to be rather in the methods section. Furthermore, we have already mentioned/cited the datasets and the time period considered (see lines 123-124 in the intro). We have now added a mention on the spatial and temporal averaging at the end of the introduction before we start describing the results.

2. Lines 109-113 – Earth system models do not fully represent all land-atmosphere interactions. Also these endeavors are not just limited by the computational difficulty to finely represent these complex biophysical effects at kilometer scales.

We have rephrased this part. We agree our statement was too optimistic regarding the capacity of models

3. Line 225 – layer and to the increased bulk … of the forests. This sentence is not clear.

We have rephrased it.

4. Lines 309 – 312 – Ths sentence is unclear.

We tried to rephrase it. Hopefully it is clearer

5. Figure 1, last line – change ‘were no’ to ‘where no’.

Corrected

6. Line 258 – change ‘that C3’ to ‘than C3’.

Corrected

7. Line 264 – change ‘an heterogeneous’ to ‘a heterogeneous’.

Corrected

8. Line 284 – change ‘value of forest’ to ‘value of forests’.

Corrected

9. Line 400 – change ‘assumption it that’ to ‘assumption is that’.

Corrected

Reviewer comments, third round –

Reviewer #3 (Remarks to the Author):

I have read through the author replies and their revisions to the manuscript. My major comment on the analysis of processes has been sufficiently addressed by the authors by moving the text to more appropriate locations rather than keeping it completely in the main text. The other comments have been addressed as well. Hence, in its current form I recommend publication of the manuscript. Best wishes to the authors for further investigations on this topic.

Regards

Point-by-point response

- Line 215. I do not understand what is meant by "passing the filtering by varying management".

Response: We rearranged and rephrased the sentence for clarification.

Other than that, it is interesting to see that the positive relationship between plant species richness and plant P stocks in the diversity experiment becomes negative in the agricultural grasslands, and that this is not caused by the agricultural grasslands having lower diversity of a few highly competitive species, as I suggested. However, the explanation involving plant biomass is still not entirely clear to me. I can follow that plant biomass is related to plant P stocks, and that because management intensity increased plant biomass, it also increased plant P stocks. That would be the blue direct arrow in Fig. 1b. I can also understand that high management intensity lowers species richness, but then why is lower species richness resulting in higher P stocks? Your explanation in the rebuttal letter suggests that fertilization effects overrule diversity effects, but would such fertilization effects not already be captured by the direct arrow in Fig. 1b between management intensity and plant P stocks? The model explicitly teases out the relationship mediated by plant species richness. My point is that effects of missing variables (such as fertilization) should be captured by the direct path, unless the missing variable is highly correlated with plant species richness, and similarly responds negatively to management intensity and itself negatively relates to plant P stocks. In short, you convinced me that my hypothesized explanation is not valid, but you have not convinced me yet of any potential mechanism behind the pattern you observe here.

Response: We rearranged and extended the explanation of the negative relationship between plant species richness and plant P stocks in the agricultural grasslands (see lines 213-227 in revised version). Notably, we stick to the perspective used for the argumentation in this paragraph namely the focus on low management intensity and associated high plant species richness. We argue that there are direct relationships between management intensity and plant P stocks likely mediated via a decrease in biomass e.g., due to lower fertilization rates. In addition, low management intensity is associated with high plant species richness and thus, the coexistence of plant species with low biomass and low nutrient concentrations. Accordingly, there is also an indirect effect of management via plant species richness that is visible in the negative relationship between plant species richness and plant P stocks. This path represents the management-induced filtering effect on plant species richness.

In other words, in the experiment, increasing species richness increases plant biomass because of complementarity and facilitation, but the plant communities were randomly composited resulting in a similar likelihood for species with high and low tissue P concentrations at all species-richness levels. In managed grasslands, however, management intensity increases specifically remove those plants that are adapted to low nutrient supply and show low biomass and low tissue P concentrations. As a consequence, increasing biodiversity increasingly involves P-poor plant species with a resulting negative effect on the P stocks in plant biomass.

- Line 478. I appreciate your explanation as to why you decided to use averages across both years. You could actually use the information you provide in the rebuttal letter that variables were significantly correlated among both years to argue why you used averages. To me, the fact that they are correlated is an indication that year-to-year variation is not huge and therefore data can be collapsed into a single average over time. I leave this decision to the authors.

Response: We inserted the information on correlations between the two years (lines 330-332 in revised version).

- Table S2 and S3. I suggested earlier to change “biomass” to “plant biomass” for clarity, and the authors say they did so, but it still seems to show as just “biomass” in my copy of the revised manuscript.

Response: Sorry for this lapse, we added plant biomass in the first row but not in the first column. We now corrected this.
